# High-Index Faceted Nanocrystals as Highly Efficient Bifunctional Electrocatalysts for High-Performance Lithium–Sulfur Batteries

**DOI:** 10.1007/s40820-021-00769-2

**Published:** 2021-12-23

**Authors:** Bo Jiang, Da Tian, Yue Qiu, Xueqin Song, Yu Zhang, Xun Sun, Huihuang Huang, Chenghao Zhao, Zhikun Guo, Lishuang Fan, Naiqing Zhang

**Affiliations:** 1 State Key Laboratory of Urban Water Resource and Environment, School of Chemistry and Chemical Engineering, Harbin Institute of Technology, Harbin, 150001 People’s Republic of China; 2 Academy of Fundamental and Interdisciplinary Sciences, Harbin Institute of Technology, Harbin, 150001 People’s Republic of China; 3 School of Energy Science and Engineering, Harbin Institute of Technology, Harbin, 150001 People’s Republic of China

**Keywords:** High-index faceted, Fe_2_O_3_ nanocrystals, Unsaturated coordinated, Lithium–sulfur batteries, Electrocatalysis

## Abstract

Precisely regulating of the surface structure of crystalline materials to improve their catalytic activity for lithium polysulfides is urgently needed for high-performance lithium–sulfur (Li–S) batteries. Herein, high-index faceted iron oxide (Fe_2_O_3_) nanocrystals anchored on reduced graphene oxide are developed as highly efficient bifunctional electrocatalysts, effectively improving the electrochemical performance of Li–S batteries. The theoretical and experimental results all indicate that high-index Fe_2_O_3_ crystal facets with abundant unsaturated coordinated Fe sites not only have strong adsorption capacity to anchor polysulfides but also have high catalytic activity to facilitate the redox transformation of polysulfides and reduce the decomposition energy barrier of Li_2_S. The Li–S batteries with these bifunctional electrocatalysts exhibit high initial capacity of 1521 mAh g^−1^ at 0.1 C and excellent cycling performance with a low capacity fading of 0.025% per cycle during 1600 cycles at 2 C. Even with a high sulfur loading of 9.41 mg cm^−2^, a remarkable areal capacity of 7.61 mAh cm^−2^ was maintained after 85 cycles. This work provides a new strategy to improve the catalytic activity of nanocrystals through the crystal facet engineering, deepening the comprehending of facet-dependent activity of catalysts in Li–S chemistry, affording a novel perspective for the design of advanced sulfur electrodes.

**Supplementary Information:**

The online version contains supplementary material available at 10.1007/s40820-021-00769-2.

## Highlights

- High-index faceted Fe_2_O_3_ nanocrystals with abundant unsaturated Fe sites not only have strong adsorption capacity to anchor LiPSs but also have superior catalytic activity to facilitate the redox conversion of LiPSs and reduce the decomposition energy barrier of Li_2_S.
- Our work deepens the comprehending of facet-dependent activity of catalysts in Li–S chemistry and affords a novel perspective for the design of advanced sulfur electrodes.

**Supplementary Information:**

The online version contains supplementary material available at 10.1007/s40820-021-00769-2.

## Graphical abstract

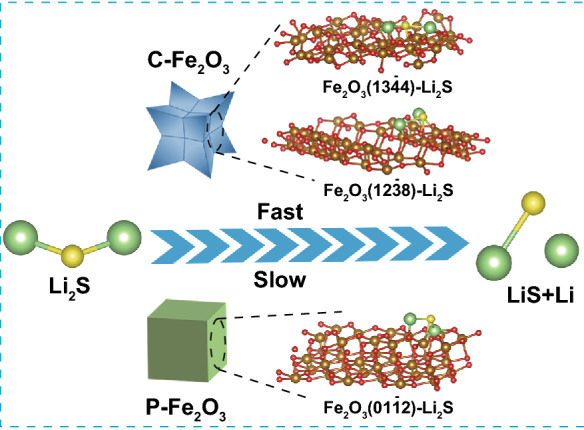

## Introduction

The booming progress of luggable electronic devices and electric vehicles urgently needs electrochemical energy storage equipment with higher power density and lower cost than lithium-ion batteries (LIBs) [1–4]. Lithium–sulfur (Li–S) batteries with high theoretical capacity (1672 mAh g^−1^), remarkable energy density (2600 Wh kg^−1^) and low cost, as one of the most promising substitutes to the current LIBs, have attracted widespread attention and ever-increasing research enthusiasm [1, 5–8]. However, the commercial application of Li–S batteries is still constrained by many challenges. The inevitable dissolution of lithium polysulfides (LiPSs) intermediates in the electrolytes and the shuttling of LiPSs between the cathode and anode result in low sulfur utilization, swift capacity degradation and the corrosion of lithium anode [9–16]. In addition, the sluggish redox kinetics resulting from insulating sulfur and Li_2_S during discharge/charge cycles limits the efficient conversion of sulfur species, impairing the rate performance and cycling stability of Li–S batteries [17–22]. Consequently, various materials have been developed and applied to Li–S batteries to tackle aforementioned issues, such as carbon matrix materials [23, 24], metal oxides [3, 25–27], metal nitrides [28, 29] and metal sulfides [30–32].

Although constructing these host materials improves the electrochemical performance of Li–S batteries to a certain extent, most of the existing researches mainly dedicated on the screening of catalytic materials which can anchor and reversibly transform LiPSs, as well as optimizing electrode materials by designing the composition, microstructure and electronic structure of the bulk phase. It is well known that both the adsorption of LiPSs and the catalytic conversion of sulfur species occur on the surfaces of electrode material [33, 34]. The surface structure of the electrode materials will directly affect the adsorption and catalytic conversion of sulfur species. However, this significant crystal facet effect in the sulfur electrochemistry has been neglected and the corresponding researches have not been reported so far. The different crystal facets exposed on electrode materials have disparate atomic arrangement [35–38], especially the high-index crystallographic planes with high densities of periodic atom steps, ledges and unsaturated coordinated sites [39–43], which may effectively regulate the adsorption and catalytic transformation of sulfur species.

Herein, the crystal facet effect in Li–S electrochemistry was systematically investigated by the design and synthesis of the high-index facet nanocrystals based on a series of electrochemical experiments and density functional theory (DFT) calculations. This work exhibited profound insight into the structure–activity relationship between the surface structures of crystal materials and the redox kinetics for sulfur species, which provided more direct theoretical basis for the rational design of advanced Li–S electrode materials in the future. In view of the low cost and nontoxicity [44–46], Fe_2_O_3_ nanocrystals with different facets were fabricated on the reduced graphene oxide (Fe_2_O_3_-G) for investigating the crystal facet effect. The theoretical and experimental results demonstrated that concave Fe_2_O_3_ nanocubes (C-Fe_2_O_3_) bounded by high-index {12$\overline{3}$8}and {13$\overline{4}$4}facets not only manifested superior inhibition effect on the shuttle of polysulfides but also had more robust catalytic activity for the transformation of sulfur species than Fe_2_O_3_ pseudocubes (P-Fe_2_O_3_) enclosed by {01$\overline{1}$2} facets. The abundant unsaturated coordinated Fe sites on the high-index faceted C-Fe_2_O_3_ as the active centers boosted the chemical adsorption for LiPSs, accelerated polysulfides conversion, in particular, enhanced the decomposition kinetics of Li_2_S, significantly improving the rate performance and cycle stability of Li–S batteries. With C-Fe_2_O_3_-G as the bifunctional electrocatalyst, the assembled batteries delivered a high initial specific capacity of 1521 mAh g^−1^ at 0.1 C and long-term cycle stability with a low capacity attenuation rate of 0.025% each cycle for 1600 cycles at 2 C. Moreover, a prominent areal capacity of 7.61 mAh cm^−2^ was maintained under a high sulfur loading of 9.41 mg cm^−2^ after 85 cycles at 0.2 C.

## Experimental Section

### Synthesis of C-Fe_2_O_3_-G, P-Fe_2_O_3_-G and Reduced Graphene Oxide

Graphite oxide was synthesized from natural graphite flakes employing a modified Hummers method. Firstly, a graphene oxide (GO) colloidal solution of 5 mg mL^−1^ was fabricated via a sonication treatment for one hour with 40 mg of GO in 8 mL of deionized water. In a typical synthesis of C-Fe_2_O_3_-G, 1.616 g of Fe(NO_3_)_3_·9H_2_O and 0.2 g of copper acetate were dissolved in deionized water (12 mL) under magnetic stirring. After twenty minutes, the homogeneous solution was mixed with above-mentioned 8 mL of GO colloidal solution and continued to be stirred for one hour in ambient atmosphere. Then 20 mL of ammonia solution (25 wt%) was quickly added into these mixtures under vigorous stirring and keep stirring for ten minutes. Finally, the resulting mixtures were transferred into a 100-mL Teflon-lined stainless steel autoclave and hydrothermally reacted at 160 °C for sixteen hours. After cooling down to room temperature naturally, the products were collected by centrifugation and washed with deionized water for several times, and subsequently freeze-dried at −50 °C to obtain C-Fe_2_O_3_-G powder. In the same experimental process, P-Fe_2_O_3_-G was synthesized by replacing copper acetate with nickel acetate and reduced graphene oxide were synthesized without the addition of metal salts.

### Material Characterization

The morphologies, structures and elemental estimation of the as-prepared products were performed via the transmission electron microscopy (TEM, FEI Tecnai G2 F30) and scanning electron microscopy (SEM, Hitachi SU8010). The crystal structures of the as-prepared materials were characterized by X-ray diffractometer (PANalytical X’Pert PRO) with Cu Kα radiation (40 mA, 40 kV). The contents of different components were measured by a thermogravimetric analyzer system (Linseis STA PT 1600). The specific surface areas and pore size distribution of the synthesized materials was explored by the Brunauer–Emmett–Teller (BET) method (using ASAP 2020, Micromeritics). X-ray photoelectron spectroscopy (XPS) measurements were taken on Thermo Scientific X-ray photoelectron spectrometer. The UV–Vis analysis was conducted with a Shimadzu UV-2450 spectrophotometer.

More details of other syntheses and characterizations can be seen in Supporting Information.

## Results and Discussion

### Characterization of C-Fe_2_O_3_-G and P-Fe_2_O_3_-G

**Fig. 1:**
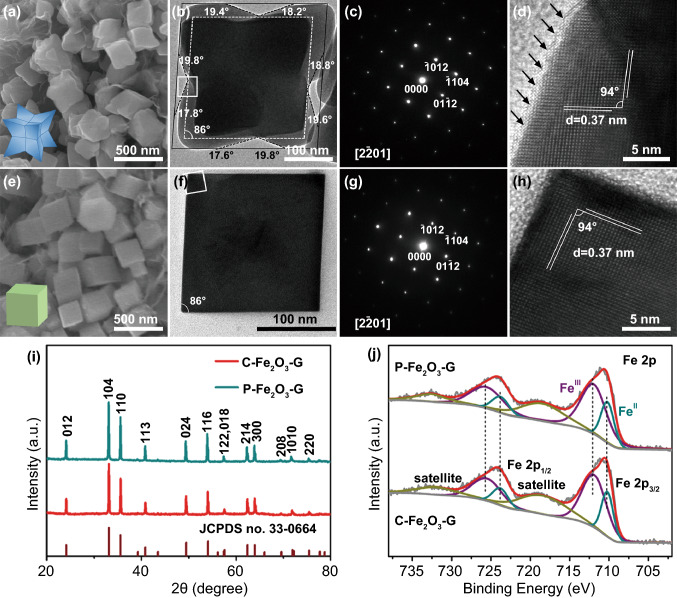
**a** SEM image, **b** TEM image, **c** corresponding SAED pattern and **d** HRTEM image of C-Fe_2_O_3_-G. **e** SEM image, **f** TEM image, **g** corresponding SAED pattern and **h** HRTEM image of P-Fe_2_O_3_-G. **i** XRD patterns and **j** Fe 2p XPS spectrums of C-Fe_2_O_3_-G and P-Fe_2_O_3_-G. The insets in **a** and **e** are the schematic models of C-Fe_2_O_3_ and P-Fe_2_O_3_, respectively

C-Fe_2_O_3_ and P-Fe_2_O_3_ with different exposed facets supported on the conductive reduced graphene oxide were synthesized by a template-free hydrothermal strategy. The reduced graphene oxide (*G*) interspersed among nanocrystals not only served for a favorable conductive network but also acted as growth matrix to block the aggregation of Fe_2_O_3_ nanocrystals. The morphology and structure characterizations of C-Fe_2_O_3_-G and P-Fe_2_O_3_-G are shown in Figs. 1 and S1-S3. As shown in the scanning electron microscope (SEM) images (Fig. S1), the dispersity of C-Fe_2_O_3_-G and P-Fe_2_O_3_-G is almost identical, and C-Fe_2_O_3_ and P-Fe_2_O_3_ were both uniformly interspersed on G. The higher-resolution SEM images (Fig. 1a, e) showed that the sizes of C-Fe_2_O_3_ and P-Fe_2_O_3_ were similar, and both average edge lengths (the distance between adjacent corners) of two kinds of nanocrystals were estimated to be approximately 300 nm. However, the morphological difference between C-Fe_2_O_3_ and P-Fe_2_O_3_ was obvious. The careful observation of the individual Fe_2_O_3_ nanocrystals at different tilting angles clearly reveals that the shape of C-Fe_2_O_3_ was concave nanocube (Fig. S2) while the shape of P-Fe_2_O_3_ was pseudocubic (Fig. S3). The reason that the shape of P-Fe_2_O_3_ was called as pseudocubic is that its dihedral angles between adjacent flat facets were 94° or 86° (Fig. S3a) [38].

In order to accurately confirm the morphology of C-Fe_2_O_3_ and P-Fe_2_O_3_ and analyze their exposed crystal facets, transmission electron microscope (TEM) was employed to characterize their fine structure and surface features. The TEM image of a single C-Fe_2_O_3_ observed along the [2$\overline{2}$01] direction is shown in Fig. 1b, and the direction of observation was confirmed by the matching select area electron diffraction (SAED) pattern (Fig. 1c), which indicated the single-crystalline nature of C-Fe_2_O_3_ [38, 47, 48]. A darker contrast in the middle of the nanocrystal than at its margins is obviously viewed in Fig. 1b, verifying the formation of concave structures. This phenomenon was not shown in TEM images of monocrystalline P-Fe_2_O_3_ observed along same direction (Fig. 1f, g), which was consistent with the formation of pseudocubic enclosed by six equivalent {01$\overline{1}$2} facets (Fig. S4a). In particular, the top view of C-Fe_2_O_3_ projected along the [2$\overline{2}$01] direction was shown as an approximate concave octagon, which was further proved the formation of the concave surfaces. In comparison with the quadrangular projection drawing of P-Fe_2_O_3_ (Fig. 1f**)**, the projected concave octagon of C-Fe_2_O_3_ could perfectly encircle a smaller quadrilateral as marked by the white dotted line in Fig. 1b. In addition, as shown in the high-resolution TEM (HRTEM) image acquired from a concave edge of C-Fe_2_O_3_ (Fig. 1d), the interplanar spacings of crossed lattice fringes with an intersection angle of 94° were all 0.37 nm, which corresponded well to the two adjacent (01$\overline{1}$2) and ($\overline{1}$012) facets exposed on an individual P-Fe_2_O_3_ (Fig. 1h) [49–51]. Based on the above analysis, the concave nanocube could be evolved from the pseudocubic [50], and then, the exposed crystal planes of C-Fe_2_O_3_ were indexed as {13$\overline{4}$4} and {12$\overline{3}$8} facets by a series of analyses with the help of geometric models and theoretical formulas (Fig. S4b).

The X-ray powder diffraction (XRD) analysis of C-Fe_2_O_3_-G and P-Fe_2_O_3_-G clearly showed that all of the diffraction peaks could be indexed to α-Fe_2_O_3_ (JCPDS no. 33–0664), which indicated that both of them not only had a high purity but also belonged to the same space group (Fig. 1i). Due to the reduction of GO to G, the characteristic diffraction peak at 10.4° of GO was not detected in C-Fe_2_O_3_-G and P-Fe_2_O_3_-G (Fig. S5) [52, 53]. These component identification results were in agreement with the Raman spectra analysis (Fig. S6). The mass ratios of G to oxide in C-Fe_2_O_3_-G and P-Fe_2_O_3_-G were investigated by thermogravimetric analysis (TGA), and the TG curves of them are shown in Fig. S7. The slight weight loss in the initial phase was due to the elimination of the absorbed water on these nanocomposites. Then the significant weight loss appeared on two TG curves, indicating oxidative decomposition of G [53]. Apparently, the mass ratios of G in C-Fe_2_O_3_-G and P-Fe_2_O_3_-G were equivalent and the G contents in two composites were both approximately 10.9 wt %, which was consistent with the theoretical calculation value. The Brunauer–Emmett–Teller (BET) specific surface areas of C-Fe_2_O_3_-G and P-Fe_2_O_3_-G were calculated using N_2_ adsorption–desorption isotherms, which were 19.71 m^2^ g^−1^ and 20.16 m^2^ g^−1^ (Fig. S8). Based on the above analysis and exclude unimportant factors, the crystal facet effect on sulfur species between C-Fe_2_O_3_-G and P-Fe_2_O_3_-G could be researched systematically.

In general, the adsorption properties and catalytic activity of materials are closely related to their surface environment (chemical composition, element valence and structural characteristics) [33, 37, 40, 49, 54, 55]. X-ray photoelectron spectroscopy (XPS) survey spectrums of C-Fe_2_O_3_-G and P-Fe_2_O_3_-G confirmed similar elemental compositions, while the states of some constituent elements are different. The high-resolution Fe 2p spectrums of C-Fe_2_O_3_-G and P-Fe_2_O_3_-G both displayed two major peaks at 711 and 724.4 eV (Fig. 1j), ascribed to the typical Fe 2p_3/2_ and Fe 2p_1/2_ orbitals, respectively [49, 52]. Each major peak could be decomposed into two fitted Gaussian components, corresponding to Fe^3+^ and Fe^2+^ species, respectively [56, 57]. The Fe^2+^ /Fe^3+^ ratio of C-Fe_2_O_3_-G was estimated to be higher than P-Fe_2_O_3_-G based on the integrated areas of the Fe^2+^ 2p_3/2_ and Fe ^3+^ 2p_3/2_ peaks. It indicated that there are more unsaturated coordinated Fe sites existed on the surface of C-Fe_2_O_3_ with abundant step atoms (the surface atom arrangements of different crystal planes are shown in Figs. 1d, h and S9) [58]. Corresponding to the theoretical simulation (Fig. S10), the coordination analysis of Fe atoms on different crystallographic planes revealed that Fe_2_O_3_ (13$\overline{4}$4) and (12$\overline{3}$8) facets possessed more unsaturated coordinated Fe atoms with dangling bonds in comparison with Fe_2_O_3_ (01$\overline{1}$2) facet.

### Comparison of LiPS Adsorption

**Fig. 2:**
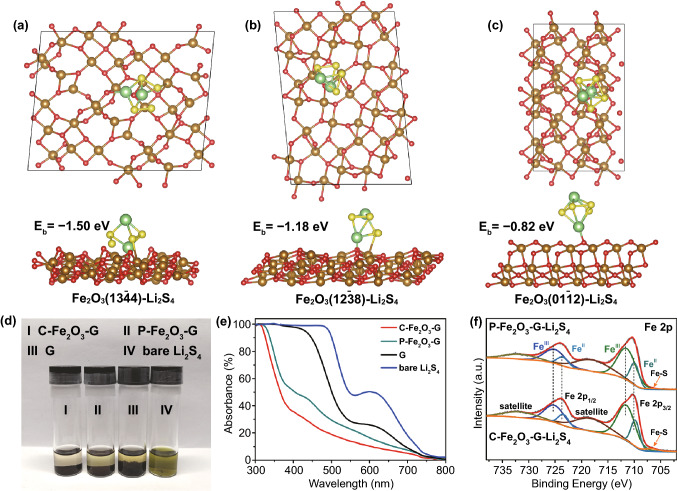
**a**–**c** Optimized geometries of Li_2_S_4_ adsorbed on different Fe_2_O_3_ crystal facets. **d** Optical photograph and **e** UV–Vis spectrums of a bare Li_2_S_4_ solution and the Li_2_S_4_ solutions with different materials after static adsorption for 5 h. **f** Fe 2p XPS comparative analysis of C-Fe_2_O_3_-G and P-Fe_2_O_3_-G after interacting with Li_2_S_4_

It is well known that the chemical adsorption of LiPSs is the precondition for their further conversion reactions on the electrocatalyst [33]. Benefiting from the unstable high-index facets with abundant unsaturated coordinated Fe sites, C-Fe_2_O_3_-G should have a stronger adsorption capacity for LiPSs than P-Fe_2_O_3_-G. DFT calculations were executed to appraise the ability to anchor LiPSs of C-Fe_2_O_3_-G and P-Fe_2_O_3_-G (Fig. 2a–c). The binding energies (*E*_b_) between different exposed crystal facets of two kinds Fe_2_O_3_ and Li_2_S_4_ were remarkably different, calculated via a formula (*E*_b_ = *E*_Li2S4**+**crystal facet_ − *E*_crystal facet_ − *E*_Li2S4_) [59]. The binding energy values of Li_2_S_4_ on the (13$\overline{4}$4) and (12$\overline{3}$8) facets of C-Fe_2_O_3_ were **−**1.50 and −1.18 eV, which were more negative than on the (01$\overline{1}$2) facets of P-Fe_2_O_3_ (**−**0.82 eV). Therefore, C-Fe_2_O_3_-G could anchor Li_2_S_4_ more efficiently than P-Fe_2_O_3_-G, owing to the more negative binding energy value signifies a stronger immobilizing effect toward Li_2_S_4_ [9, 28, 29].

To further validate the stronger interaction between C-Fe_2_O_3_-G and LiPSs, the visualized adsorption experiments were performed by adding C-Fe_2_O_3_-G or P-Fe_2_O_3_-G or G with the same mass into the Li_2_S_4_ solution. As shown in Fig. 2d, the Li_2_S_4_ solution with C-Fe_2_O_3_-G was almost completely faded after static adsorption for 5 h, whereas the Li_2_S_4_ solutions containing P-Fe_2_O_3_-G or G still remained gradually deepened yellow. The UV/Vis spectrums of these solutions after aging exhibited that more Li_2_S_4_ were adsorbed by C-Fe_2_O_3_-G than others, indicating a superior binding capability toward LiPSs of C-Fe_2_O_3_-G than P-Fe_2_O_3_-G and G (Fig. 2e). The visualized adsorption experiments of Li_2_S_6_ displayed the same results (Fig. S11), which further visually demonstrated the superiority of C-Fe_2_O_3_-G in adsorbing LiPSs. In addition, XPS analyses of C-Fe_2_O_3_-G and P-Fe_2_O_3_-G dried after Li_2_S_4_ adsorption (C-Fe_2_O_3_-G-Li_2_S_4_ and P-Fe_2_O_3_-G-Li_2_S_4_) were performed to further reveal the chemical interaction toward Li_2_S_4_ between C-Fe_2_O_3_-G and P-Fe_2_O_3_-G (Fig. 2f). In comparison with pristine nanocomposites without Li_2_S_4_, the four characteristic peaks of Fe^2+^ 2p_3/2_, Fe^2+^ 2p_1/2_, Fe^3+^ 2p_3/2_ and Fe^3+^ 2p_1/2_ overall shifted toward lower binding energies (Fig. 2f), implying the strong chemical interaction between Li_2_S_4_ and two nanocomposites [60]. Since the four characteristic peaks of C-Fe_2_O_3_-G located at the same position as those corresponding characteristic peaks of P-Fe_2_O_3_-G as shown in Figs. 1j and S12, it was easy to observe the shift gaps of Fe^2+^ 2p peaks between C-Fe_2_O_3_-G-Li_2_S_4_ and P-Fe_2_O_3_-G-Li_2_S_4_ (Fig. 2f). These shift gaps indicated the stronger chemical interaction between Fe^2+^ 2p sites on C-Fe_2_O_3_-G and Li_2_S_4_ than those on P-Fe_2_O_3_-G, which was attributed to the more Fe^2+^ sites that have interacted with S_x_^2−^ on the surface of C-Fe_2_O_3_-G, namely the exposed high-index {12$\overline{3}$8} and {13$\overline{4}$4} crystal facets on C-Fe_2_O_3_-G provided more unsaturated coordinated Fe^2+^ sites, leading to the more effective bonding of S_x_^2−^. Noteworthy, a distinct additional peak representative of Fe–S bond was appeared at 706.7 eV in Fe 2p spectrum of C-Fe_2_O_3_-G-Li_2_S_4_, while this characteristic peak of Fe–S bond in Fe 2p spectrum of P-Fe_2_O_3_-G-Li_2_S_4_ was significantly smaller (Fig. 2f), which further confirmed the stronger Fe–S interactions between C-Fe_2_O_3_-G and Li_2_S_4_ than P-Fe_2_O_3_-G [52, 57]. Furthermore, the stronger Fe–S bond and more obvious forward movement of the terminal sulfur (S_T_^−1^) were showed in S 2p XPS spectrum of C-Fe_2_O_3_-G-Li_2_S_4_ compared with those of P-Fe_2_O_3_-G-Li_2_S_4_, which also demonstrated the stronger adsorption capacity of C-Fe_2_O_3_-G for LiPSs (Fig. S 13) [26].

### Evaluation of Catalytic Activity

**Fig. 3:**
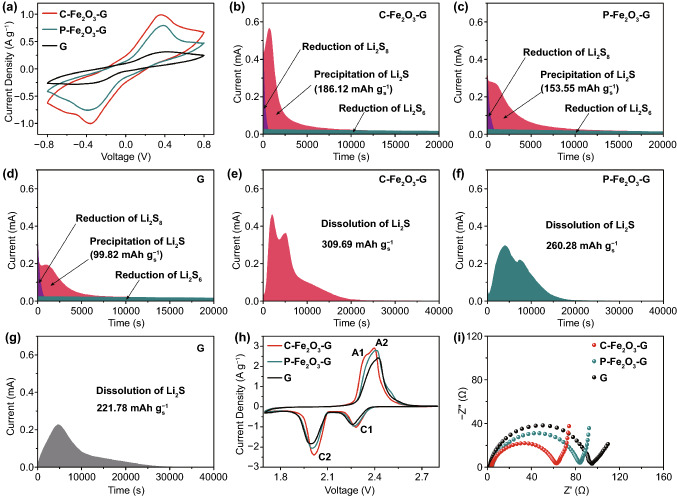
**a** CV curves of the symmetric batteries with C-Fe_2_O_3_-G, P-Fe_2_O_3_-G and G electrodes. Potentiostatic discharge profiles of Li_2_S nucleation on **b** C-Fe_2_O_3_-G, **c** P-Fe_2_O_3_-G and **d** G. Potentiostatic charge profile of Li_2_S dissolution on **e** C-Fe_2_O_3_-G, **f** P-Fe_2_O_3_-G and **g** G. **h** CV curves and **i** corresponding Nyquist plots of asymmetrical batteries with C-Fe_2_O_3_-G, P-Fe_2_O_3_-G and G

In order to gain insight into the efficacy of high-index faceted catalysts in accelerating the liquid–liquid conversion of LiPSs, the redox reaction kinetics of LiPSs were systemically analyzed by cyclic voltammetry (CV) experiments for the Li_2_S_6_ symmetric batteries (Fig. 3a), which were assembled by sandwiching commercialized polypropylene (PP) membrane between two same sulfur-free electrodes and filled with Li_2_S_6_ electrolyte. Obviously, the CV curve of Li_2_S_6_ symmetric battery with C-Fe_2_O_3_-G electrodes displayed a higher polarization current than those with P-Fe_2_O_3_-G and G electrodes under a scan rate of 5 mV s^−1^, implying that C-Fe_2_O_3_-G had significantly stronger effectiveness in enhancing the redox kinetics between liquid-phase LiPSs [59].

In comparison with the liquid–liquid conversion of LiPSs, the liquid–solid–liquid conversion involving the nucleation and decomposition of Li_2_S could control the sulfur utilization and specific capacity of Li–S batteries more effectively [61]. Therefore, it was important to evaluate the catalytic effect for the deposition and dissolution of Li_2_S by catalyst material [61]. To investigate the Li_2_S precipitation process, simple potentiostatic discharge experiments of C-Fe_2_O_3_-G, P-Fe_2_O_3_-G and G electrodes were executed (Fig. 3b–d). The C-Fe_2_O_3_-G electrode took less time to reach a higher current peak of 0.57 mA than P-Fe_2_O_3_-G electrode and G electrode under 2.05 V. Meanwhile, the capacity of Li_2_S precipitation on C-Fe_2_O_3_-G (186.12 mAh g^−1^) was higher than those on P-Fe_2_O_3_-G (153.55 mAh g^−1^) and G (99.82 mAh g^−1^). The above results demonstrated that C-Fe_2_O_3_-G could markedly facilitate Li_2_S nucleation and deposition amounts. In addition, the similar kinetic studies were performed via a potentiostatic decompositions after the galvanostatic discharge processes to verify the superiority of C-Fe_2_O_3_-G for boosting the dissolution of deposited Li_2_S. As shown in Fig. 3e-g, both the oxidation current density and Li_2_S dissolution capacity of C-Fe_2_O_3_-G electrode were higher compared to those of P-Fe_2_O_3_-G and G electrodes, revealing that C-Fe_2_O_3_-G could reduce the oxidation overpotential and enhance the kinetics of Li_2_S dissolution and conversion more effectively than the other two materials during charging [33, 59, 61].

### Comparison of Electrochemical Performance

To evaluate the practical superiority of C-Fe_2_O_3_-G for enhancing the electrochemical kinetics of LiPS transformation in a working Li–S battery, C-Fe_2_O_3_-G, P-Fe_2_O_3_-G and G with the same mass were coated on the commercial PP membranes to obtain the functionalized separators. In Fig. S14, C-Fe_2_O_3_-G and P-Fe_2_O_3_-G adhered evenly on the PP membrane surface to assemble the faultless interlayers with a thickness of 15 µm. And then, the accelerated redox reactions of sulfur species transformation were explored by CV measurements of Li–S batteries assembled with these functionalized separators between C–S cathodes (Fig. S15) and Li metal anodes at 0.1 mV s^−1^. The CV curves recorded within a voltage window of 1.7–2.8 V all obviously exhibited the cathodic (reduction) peaks and anodic (oxidation) peaks, which respectively corresponded to the reduction of sulfur to soluble LiPSs [29], LiPSs to Li_2_S_2_/Li_2_S and the oxidation of Li_2_S_2_/Li_2_S to sulfur (Fig. 3h). Obviously, the positive shift of two cathodic peaks to a higher voltage and the negative shift of the anodic peak to a lower voltage, as well as the enhanced current of all redox peaks were exhibited on the cell with C-Fe_2_O_3_-G interlayer (C-Fe_2_O_3_-G cell) compared to those with P-Fe_2_O_3_-G and G interlayers (P-Fe_2_O_3_-G cell and G cell), indicating that C-Fe_2_O_3_-G had more robust catalytic ability to enhance the redox kinetics of LiPSs [9–11, 33, 59]. The peculiar A1 peak appeared only on the CV curve of C-Fe_2_O_3_-G cell, which signified a rapider Li_2_S dissolution behavior on C-Fe_2_O_3_-G than others [29]. Furthermore, in comparison with P-Fe_2_O_3_-G cell and G cell, the electrochemical impedance spectroscopy (EIS) of C-Fe_2_O_3_-G cell showed the smallest charge transfer resistance, manifesting the superior interfacial charge conductivity under C-Fe_2_O_3_-G electrocatalysis, which resulted in the accelerated sulfur redox kinetics (Fig. 3i).

Subsequently, CV measurements were also implemented under higher scan rates (0.2 to 0.6 mV s^−1^) to investigate lithium-ion diffusion coefficients, which were another important impact factor for the transformation kinetics of LiPSs [61], to confirm the superior electrochemical performance of C-Fe_2_O_3_-G in LiPS conversion. All reduction and oxidation peak currents varied linearly with the square root of scanning rate, and the slopes of the curves obtained from the linear fitting of peak currents were positively interrelated with the corresponding lithium-ion diffusion in cells with different interlayers (Fig. S16). Evidently, C-Fe_2_O_3_-G cell exhibited the largest slope value in each reduction and oxidation reaction of sulfur species, which certified the superiority of C-Fe_2_O_3_-G in accelerating mass transfer and LiPS redox kinetics during discharge/charge [29, 61]. To further demonstrate the faster lithium-ion transport kinetics of the C-Fe_2_O_3_-G, the galvanostatic intermittent titration technique (GITT) was employed to analyze the lithium-ion diffusion coefficient (D_Li_^+^) in three battery systems (Fig. S17a-c). The calculated D_Li_^+^ values in C-Fe_2_O_3_-G cell were larger than those in P-Fe_2_O_3_-G and G cells (Fig. S17d), further confirming the superiority of C-Fe_2_O_3_-G in accelerating the lithium-ion transfer.

**Fig. 4:**
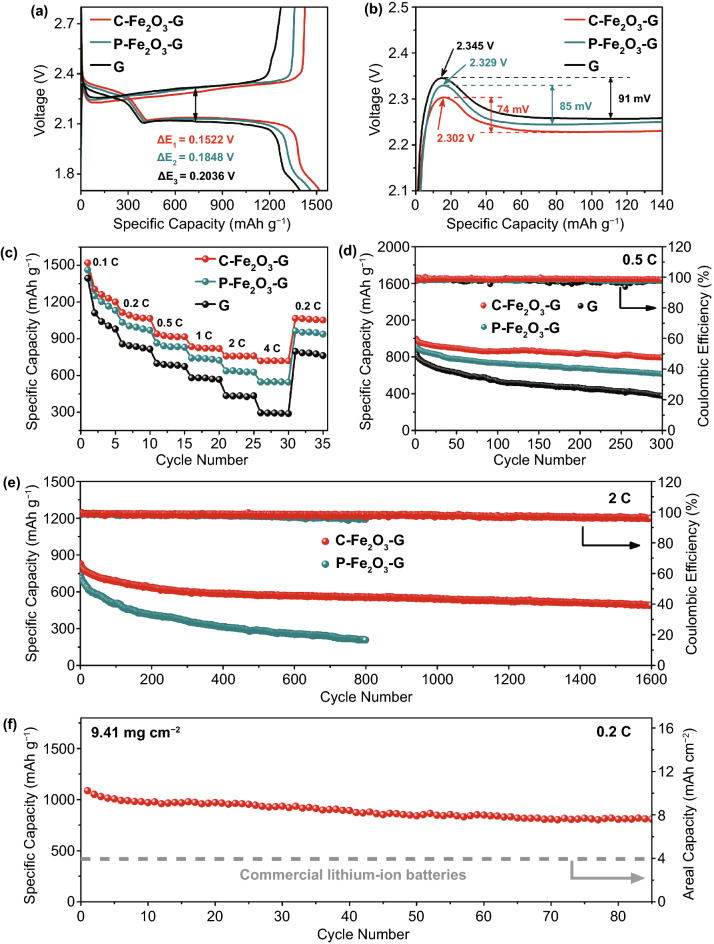
**a** Galvanostatic charge/discharge profiles and **b** charge voltage profiles of C-Fe_2_O_3_-G, P-Fe_2_O_3_-G and G cells at 0.1C. **c** Rate capacities and **d** cycle performance at 0.5 C of C-Fe_2_O_3_-G, P-Fe_2_O_3_-G and G cells. **e** Long-term cycle stability of C-Fe_2_O_3_-G and P-Fe_2_O_3_-G cells at 2 C. **f** Cycling performance of C-Fe_2_O_3_-G cell with high sulfur loading of 9.41 mg cm^−2^ at 0.2 C

The galvanostatic charge–discharge measurements of the cells with different catalytic materials under 0.1 C also revealed similar results matched with the above analyses. The charge–discharge curves within a cutoff voltage of 1.7–2.8 V showed two discharge plateaus and a charge plateau, respectively, assigning to the reduction and oxidation peaks of CV curves (Fig. 4a). In comparison with P-Fe_2_O_3_-G cell and G cell, C-Fe_2_O_3_-G cell manifested higher discharge capacity at the first voltage plateau (Q_1_) and second voltage plateau (Q_2_) as well as lager capacity ratio of Q_2_ to Q_1_, which not only indicated the superiority in suppressing the shuttling of LiPSs but also verified the stronger catalytic effect in promoting the conversion of LiPSs to unsolvable Li_2_S [59]. The cell with C-Fe_2_O_3_-G exhibited the smallest polarization (∆*E*_1_ = 0.1522 V) than those with P-Fe_2_O_3_-G (∆*E*_2_ = 0.1848 V) and G (∆*E*_3_ = 0.2036 V), further proving the enhanced electrochemical kinetics stemming from C-Fe_2_O_3_-G. In the charge process, the smallest initial charge potential barrier was displayed on the curve of C-Fe_2_O_3_-G cell, which implied a most robust catalytic effect of C-Fe_2_O_3_-G in the decomposition of Li_2_S (Fig. 4b) [33, 59].

To further validate the significant impact of C-Fe_2_O_3_-G on boosting the sulfur redox kinetics, the rate capacities of the batteries assembled with different interlayers were first evaluated under increasing current density from 0.1 to 4.0 C (Fig. 4c). C-Fe_2_O_3_-G cell exhibited the highest initial discharge capacity of 1521 mAh g^−1^ at 0.1 C among three battery systems with the sulfur loading of 1.0–1.4 mg cm^−2^. When increasing the electric current density to 0.2, 0.5, 1.0, 2.0 and 4.0 C, the reversible discharge capacities of C-Fe_2_O_3_-G cell could still reach 1115, 941, 835, 760 and 719 mAh g^−1^, respectively, which were much higher than the corresponding capacities of P-Fe_2_O_3_-G cell and G cell. Moreover, the capacity gaps between C-Fe_2_O_3_-G cell and the other two cells with P-Fe_2_O_3_-G or G gradually expanded with the increase of electric current density, respectively, attaining 172 and 424 mAh g^−1^ at 4.0 C. The corresponding charge–discharge profiles of three batteries at different current densities were recorded, as shown in Fig. S18. Even at a high current densities of 4.0 C, C-Fe_2_O_3_-G cell still maintained two well-defined discharge plateaus, exhibiting the higher electrochemical stability than two other cell systems. These test results all demonstrated that C-Fe_2_O_3_-G could not only more evidently alleviate the shuttling of LiPSs but also enhance the utilization of sulfur more effectively, which possibly profited from the excellent adsorption capacity and catalytic activity of the high-index crystal facets exposed on C-Fe_2_O_3_-G.

The enhanced cycling stability of Li–S batteries with C-Fe_2_O_3_-G catalysts was also testified via an endurance test under the galvanostatic mode. The cycle performances at 0.5 C of the batteries with C-Fe_2_O_3_-G, P-Fe_2_O_3_-G and G are shown in Fig. 4d. It was clear that C-Fe_2_O_3_-G cell delivered higher initial discharge capacities in comparison with P-Fe_2_O_3_-G cell and G cell, revealing that C-Fe_2_O_3_-G interlayer could reduce the loss of the active sulfur components most effectively [29, 59]. After continuous 300 cycles, C-Fe_2_O_3_-G cell held a high reversible capacity of 788 mAh g^−1^ with high average Coulombic efficiency (> 98.5%), corresponding to the average capacity fading of 0.069% each cycle. By contrast, P-Fe_2_O_3_-G cell and G cell, respectively, retained discharge capacities of 616 and 349 mAh g^−1^ after 300 cycles at 0.5 C, respectively, corresponding to two higher capacity decay rates. Consequently, the battery with C-Fe_2_O_3_-G catalyst showed better cycling stability under a low current rate of 0.5 C. The long-term cycling stability measurements were also taken under a higher current rate of 2.0 C, and the corresponding results are shown in Fig. 4e. C-Fe_2_O_3_-G cell exhibited a higher premier discharge capacity and much better cycling performance than P-Fe_2_O_3_-G cell. After 1600 continuous discharging–charging cycle tests, C-Fe_2_O_3_-G cell still maintained a reversible discharge capacity of 491 mAh g^−1^, achieving a capacity fading rate as low as 0.025% every cycle. Besides, the good voltage stability during the long-term charge–discharge cycles further manifested the excellent electrochemical stability of C-Fe_2_O_3_-G cell (Fig. S19). In comparison, the discharge specific capacity of P-Fe_2_O_3_-G cell decayed to 209 mAh g^−1^ after 800 continuous cycles, and the capacity fading of each cycle was 0.089%, revealing the worse long-term cycling stability than C-Fe_2_O_3_-G cell at 2.0 C. In addition, the Coulombic efficiency of C-Fe_2_O_3_-G cells had smaller decline compared with P-Fe_2_O_3_-G cells both at 0.5 and 2.0 C, implying better inhibition of C-Fe_2_O_3_-G for LiPS shuttling. The effective suppression of LiPS shuttling would guarantee the slightest corrosions of Li anode, which was identified by the characterization of Li metal anodes in Fig. S20. The long-term cycling stability of C-Fe_2_O_3_-G cell at a higher current density of 4.0 C was also recorded under the galvanostatic mode. As shown in Fig. S21, C-Fe_2_O_3_-G cell exhibited the good long-term cycling stability at 4.0 C, which further confirmed the superiority of C-Fe_2_O_3_-G in improving the performance of Li–S batteries.

The chemical stability of C-Fe_2_O_3_-G during the charge–discharge cycle was certified by XRD and XPS analyses. As shown in Fig. S22**,** all the XRD diffraction peaks of the cycled C-Fe_2_O_3_-G highly matched with the new C-Fe_2_O_3_-G. Moreover, the characteristic peak of Fe–S bond did not appear in the high-resolution Fe 2p spectrums of the cycled C-Fe_2_O_3_-G (Fig. S23). All these characterization results indicated that the sulfidation reaction of C-Fe_2_O_3_-G by sulfur species did not occur during cycling, which proved the excellent chemical stability of C-Fe_2_O_3_-G in lithium–sulfur batteries. Taking into account the high requirement of energy density in practical applications, the cycle performances of C-Fe_2_O_3_-G cell with a high sulfur loading of 9.41 mg cm^−2^ was investigated under the galvanostatic mode. The battery with C-Fe_2_O_3_-G electrocatalysts, respectively, delivered discharge capacity of 1192 and 1087 mAh g^−1^ at 0.1 and 0.2 C (Fig. S24). After 85 continuous discharging–charging cycles at 0.2 C, the cell maintained a reversible discharge capacity of 809 mAh g^−1^, corresponding to a favorable areal capacity of 7.61 mAh cm^−2^, which was much superior to the commercial LIBs. This high discharge capacity and superior rate performance as well as outstanding cycle span are prominent in comparison with previous works (Table S1). All the research results confirmed that C-Fe_2_O_3_-G with high-index crystal faces could effectively elevate the electrochemical performance of sulfur species in a working battery, which was conducive to the practical application of Li–S batteries.

### DFT Analysis

**Fig. 5:**
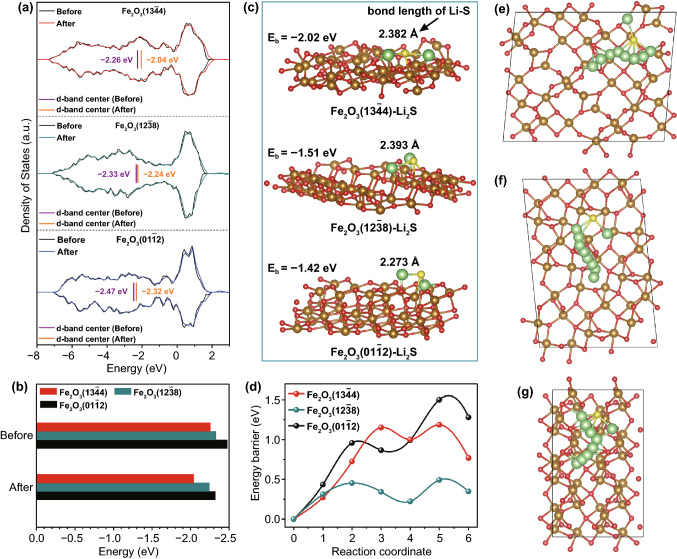
**a** Projected density of states and **b** d-band center of the Fe atoms exposed on different Fe_2_O_3_ crystal planes before and after interacting with Li_2_S_4_. **c** Optimized geometries and **d** decomposition energy barriers of Li_2_S adsorbed on different Fe_2_O_3_ crystal faces. **e**–**g** Li_2_S decomposition path on Fe_2_O_3_ (13$\overline{4}$4), (12$\overline{3}$8) and (01$\overline{1}$2) facets. The Li, S, Fe and O atoms are severally indicated by green, yellow, gold and red balls

The DFT calculations were carried out to further uncover the mechanism of C-Fe_2_O_3_-G electrocatalysts more efficiently inhibiting the shuttling of LiPSs and accelerating the redox kinetics of sulfur species. The projected density of states (PDOS) of Fe-3d orbitals for different crystal facets before and after interacting with Li_2_S_4_ is shown in Fig. 5a, revealing the electronic configuration of Fe center, which was related to surface adsorbability and catalytic activity of Fe-based catalysts [59]. In Fig. 5b, the d-band centers of Fe atoms on the (13$\overline{4}$4) (−2.26 eV) and (12$\overline{3}$8) (−2.33 eV) facets of C-Fe_2_O_3_ were closer toward the Fermi level than those on Fe_2_O_3_ (01$\overline{1}$2) facet (−2.47 eV), indicating the stronger adsorption capacity and better electronic conductivity on Fe_2_O_3_ (13$\overline{4}$4) and (12$\overline{3}$8) facets, which would be beneficial to the adsorption and further conversion of LiPSs [9, 59, 62]. After interacting with LiPSs, the d-band center of Fe atoms on Fe_2_O_3_ (13$\overline{4}$4) facet shifted more obviously to the Fermi level compared to those on the other two crystal facets, corresponding to the strongest interaction between catalyst surface and adsorbates [63]. Li_2_S_4_ adsorbed on Fe_2_O_3_ (13$\overline{4}$4) facet displayed an unusual distorted geometry with broken Li–S and S–S bond (Fig. 2a), reflecting that Fe_2_O_3_ (13$\overline{4}$4) facets could adsorb and activate Li_2_S_4_ more effectively and expedite the decomposition of Li_2_S_4_ [29]. In addition, the adsorption of Li_2_S on different Fe_2_O_3_ crystal faces was analyzed by DFT calculations (Figs. 5c and S25). The calculated binding energies of Li_2_S on Fe_2_O_3_ (13$\overline{4}$4) and (12$\overline{3}$8) facets were −2.02 and −1.51 eV, respectively, which were more negative in comparison with that on Fe_2_O_3_ (01$\overline{1}$2) facet (−1.42 eV), availing more uniform Li_2_S nucleation and deposition [59]. It is worth noting that the bond lengths of Li–S in adsorbed Li_2_S both on Fe_2_O_3_ (13$\overline{4}$4) (2.382 Å) and (12$\overline{3}$8) (2.393 Å) facets were significantly longer than that on Fe_2_O_3_ (01$\overline{1}$2) facet (2.273 Å), which suggested that C-Fe_2_O_3_ as catalysts could more efficiently weaken the binding between Li and S of Li_2_S and then reduce the decomposition energy barrier of Li_2_S [64]. Afterward, the superiority of C-Fe_2_O_3_ was further confirmed by the theoretical analysis of decomposition energy barrier of Li_2_S on different Fe_2_O_3_ crystal faces. The energy profiles of Li_2_S decomposition and corresponding decomposition path are shown in Fig. 5d–g. The decomposition energy barriers of Li_2_S on Fe_2_O_3_ (12$\overline{3}$8) (0.49 eV) and (13$\overline{4}$4) (1.18 eV) facets were significantly lower than that on Fe_2_O_3_ (01$\overline{1}$2) facet (1.51 eV), which revealed that C-Fe_2_O_3_ possessed stronger catalytic capacity to break Li–S bonds more easily and enhance the oxidative decomposition kinetics of Li_2_S [65]. These theoretical calculation results all demonstrated that the higher sulfur utilization and faster reversible conversion of sulfur species could realize with the help of C-Fe_2_O_3_-G catalysts compared with P-Fe_2_O_3_-G, which was in good agreement with electrochemical measurement results.

## Conclusions

We successfully constructed Fe_2_O_3_ concave nanocubes with high-index facets anchored on reduced graphene oxide through a simple hydrothermal strategy and applied it as the electrocatalysts to investigate the structure–activity relationship between the surface structures of crystal materials and its chemisorption/catalytic conversion for sulfur species. Experiment researches and DFT results all revealed that the strong adsorption capacity and high catalytic activity of C-Fe_2_O_3_-G stemmed from the active high-index Fe_2_O_3_ crystal faces with abundant unsaturated Fe sites. These high-activity crystal facets could not only enhance chemisorption of LiPSs but also accelerate the liquid–solid conversion of LiPSs and the oxidative decomposition of Li_2_S, which significantly improve the utilization of sulfur. Therefore, the batteries with C-Fe_2_O_3_-G catalysts delivered an outstanding premier discharge capacity of 1521 mAh g^−1^ at 0.1 C, as well as the stable cycling performance during 1600 cycles at 2C with a low capacity decaying of 0.025% every cycle. Moreover, the battery with a high sulfur loading of 9.41 mg cm^−2^ cycled steadily at 0.2 C and a high areal capacity of 7.61 mAh cm^−2^ was maintained after 85 cycles. This work exhibited pioneering insights into the crystal facet effect in Li–S electrochemistry and provided instructive guidance for fabricating novel catalysts applied in advanced Li–S batteries by tuning the surface structure of materials.

## Supplementary Information

Below is the link to the electronic supplementary material.

Supplementary file1 (PDF 2694 KB)
