# Supplementary material for: High-Index Faceted Nanocrystals as Highly Efficient Bifunctional Electrocatalysts for High-Performance Lithium–Sulfur Batteries
Source: Nanomicro Lett. 2021 Dec 23;14:40. doi: 10.1007/s40820-021-00769-2 (PMC8702595; doi:10.1007/s40820-021-00769-2)
Supplement: Supplementary file 1 — Supplementary file1 (PDF 2694 KB) [file 40820_2021_769_MOESM1_ESM.pdf]

Supporting Information for

High-Index Faceted Nanocrystals as Highly Efficient Bifunctional Electrocatalysts for High-Performance Lithium-Sulfur Batteries

Bo Jiang[1, §], Da Tian[1, §], Yue Qiu[1, §], Xueqin Song[1], Yu Zhang[3], Xun Sun[1], Huihuang Huang[1], Chenghao Zhao[1], Zhikun Guo[1], Lishuang Fan[1, 2, *], and Naiqing Zhang[1, 2, *]

[1]State Key Laboratory of Urban Water Resource and Environment, School of Chemistry and Chemical Engineering, Harbin Institute of Technology, Harbin 150001, P. R. China

[2]Academy of Fundamental and Interdisciplinary Sciences, Harbin Institute of Technology, Harbin 150001, P. R. China

[3]School of Energy Science and Engineering, Harbin Institute of Technology, Harbin 150001, P. R. China

[§]B. Jiang, D. Tian and Y. Qiu contributed equally to this work

*Corresponding authors. E-mail: znqmww@163.com (Naiqing Zhang); fanlsh@hit.edu.cn (Lishuang Fan)

S1 Supplementary Experimental Section

S1.1 Preparation of C-Fe_2O_3-G/PP, P-Fe_2O_3-G/PP and G/PP Separators

Four milligrams of C-Fe_2O_3-G, P-Fe_2O_3-G or reduced graphene oxide were mixed with one milligram of Super P separately. These mixtures were dispersed into 15 mL of N-methylpyrrolidone (NMP) respectively and sonicated for two hours to form a uniform mixture. Then these materials dispersing in solutions were coated onto the Celgard polypropylene (PP) separators separately by a vacuum filtration and then dried at 60 °C overnight in an oven. The areal masses of C-Fe_2O_3-G, P-Fe_2O_3-G and graphene on the separators are all 0.318 mg cm^{-2}.

S1.2 Li-S Batteries Assembly and Electrochemical Measurements

The sulfur-carbon composite compound with 75 wt% sulfur content (Fig. S15) was fabricated by a simple melting-diffusion method. 50 mg of Ketjen black carbon and 150 mg of sublimed sulfur were mixed together and grinded for forty minutes, followed with heating at 155 °C for twenty-four hours in a sealed Teflon-lined stainless steel autoclave. After cooling down to room temperature naturally, 90 wt% sulfur-carbon composite mixed with 10 wt% LA133 binder with the help of just enough water to obtain the sulfur slurry, which was spread on a carbon-coated aluminum foil current collector and dried at room temperature overnight. Then, the above-prepared products were punched into a disk with a diameter of 12 mm, obtaining the sulfur cathodes with a sulfur areal loading of 1.0~1.4 mg cm^{-2}. The standard 2025 coin cells were assembled with Li metal disc as the anode, as-prepared sulfur cathode and the functional PP separator with the functionalized material to investigate the electrochemical performance of different interlayers in a glovebox filled with argon. The amount of electrolyte added to the batteries varied from 22 to 30 μL depending on the loading of sulfur, maintaining an E/S (electrolyte/sulfur) ratio of 19 μL mg^{-1}. The electrolyte was composed of 1 mol L^{-1} lithium bis(trifluoromethylsulfonyl)imide (LiTFSI) in a mixed solvent of 1, 3-dioxolane (DOL) and

dimethoxymethane (DME) (v/v=1:1) with 2% $LiNO_3$ addition. With the increase of the areal loading of sulfur in cathodes (1.0~1.4 mg/cm^{-2}), the mass ratio of Fe_2O_3 to carbon (G + Super P + Ketjen black) in the cells decreased from 1.18 to 0.97, while the mass ratio of sulfur to carbon (G + Super P + Ketjen black) increased from 1.59 to 1.83. When we assembled the C-Fe_2O_3-G cell with a high sulfur loading of 9.41 mg cm^{-2}, 75 μL of electrolyte was added in it. The cyclic voltammetry (CV) measurements were recorded on a ChenHua CHI660D electrochemical workstation between 1.7 and 2.8 V. The charge transfer kinetics was explored via electrochemical impedance spectroscopy (EIS) measurements using a PARSTAT 2273 advanced electrochemical system, and the frequency range from 1 MHz to 0.1 Hz. The charge/discharge tests were carried out using the Neware battery test system in the voltage window of 1.7~ 2.8 V.

S1.3 Synthesis of LiPSs Solution

The LiPSs (Li_2S_x, x=4-8) solution was prepared by chemically reacting sublimed sulfur and lithium sulfide (Li_2S) with stoichiometry (Mole ratio of S: Li_2S was 3:1, 5:1 and 7:1) in a mixed solution of DOL/DME (1:1 by volume) with 1mol/L LiTFSI and 2% $LiNO_3$. The mixed solutions were then quickly stirred at 40 °C in a glovebox filled with argon until all the solid particles were completely dissolved, obtaining LiPSs solution.

S1.4 Assembly of Li_2S_6 Symmetric Cells and Kinetic Characterization of Polysulfide Transformation

The Li_2S_6 symmetric batteries were fabricated by sandwiching commercialized PP separator between two sulfur-free electrodes with different catalyst materials and filled with Li_2S_6 electrolyte in a glovebox filled with argon. The CV curves of the symmetric cells were recorded at different scan rates ranging from 1 to 200 mV s^{-1} with a voltage window between -0.8 and 0.8 V on the electrochemical workstation.

S1.5 Li_2S nucleation and Decomposition Tests

The batteries were assembled by sandwiching Celgard 2400 membrane between the sulfur-free electrodes with different catalyst materials and Li metal discs in a glovebox filled with argon, meanwhile, 30 μL Li_2S_8 electrolyte were added between the sulfur-free cathodes and Li metal anode. For the Li_2S nucleation and growth tests, the cells were firstly discharged galvanostatically to 2.11 V at a current density of 0.112 mA and then discharged potentiostatically at 2.05 V until the discharging current decreased to 0.01 mA. The whole charges were collected to evaluate the Li_2S nucleation/growth rate based on the Faraday's law [S1, S2]. For the Li_2S decomposition process, the cells were firstly discharge galvanostatically at a current density of 0.1 mA to 1.9 V, and then discharge galvanostatically at a current density of 0.01 mA to 1.7 V ensuring that LiPSs fully transformed to insoluble Li_2S. After completely discharged, the cells were charged potentiostatically at 2.40 V until the charging current was below 0.01 mA to realize the complete decomposition of insoluble Li_2S into soluble LiPSs.

S1.6 Theoretical Calculations

The spin-polarized density functional theory (DFT) calculations were carried out based on the Quantum ESPRESSO package [S3] with the projector augmented wave (PAW) [S4, S5] method. The Perdew-Burke-Ernzerhof (revPBE) functional [S6] of generalized gradient approximation (GGA) was used to describe the exchange correlation term. All geometrical structures were fully optimized to its ground state with a cutoff energy of 400 eV [S4, S5, S7]. The energy and forces are converged to 1×10^{-4}eV and 0.05 eV Å^{-1}, respectively. The Li^+ diffusion was simulated using the climbing image nudged elastic band (CI-NEB) method [S8].

S2 Supplementary Figures and Table

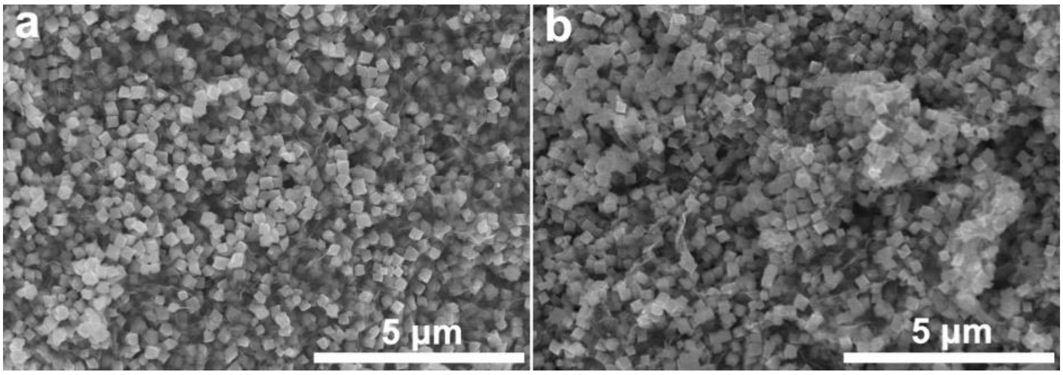

Fig. S1 SEM images of C-Fe_2O_3-G and P-Fe_2O_3-G

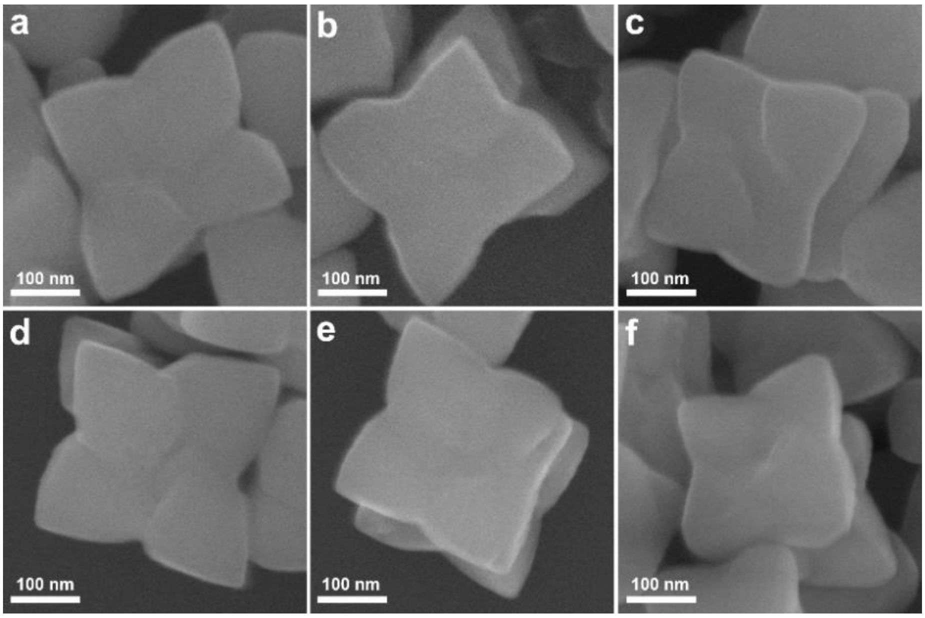

Fig. S2 High-resolution SEM images of C-Fe_2O_3 at different tilting angles

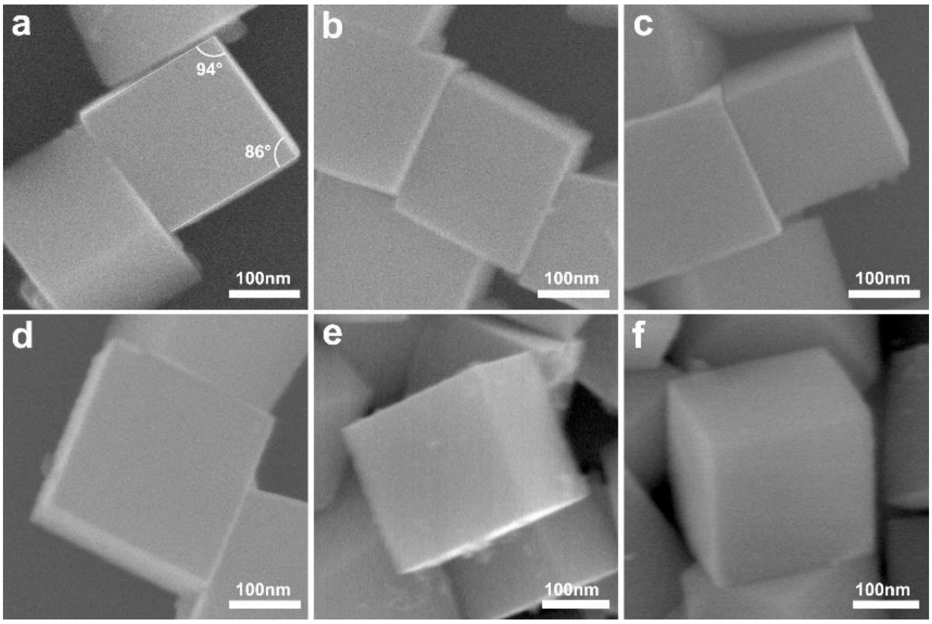

Fig. S3 High-resolution SEM images of P-Fe_2O_3 at different tilting angles

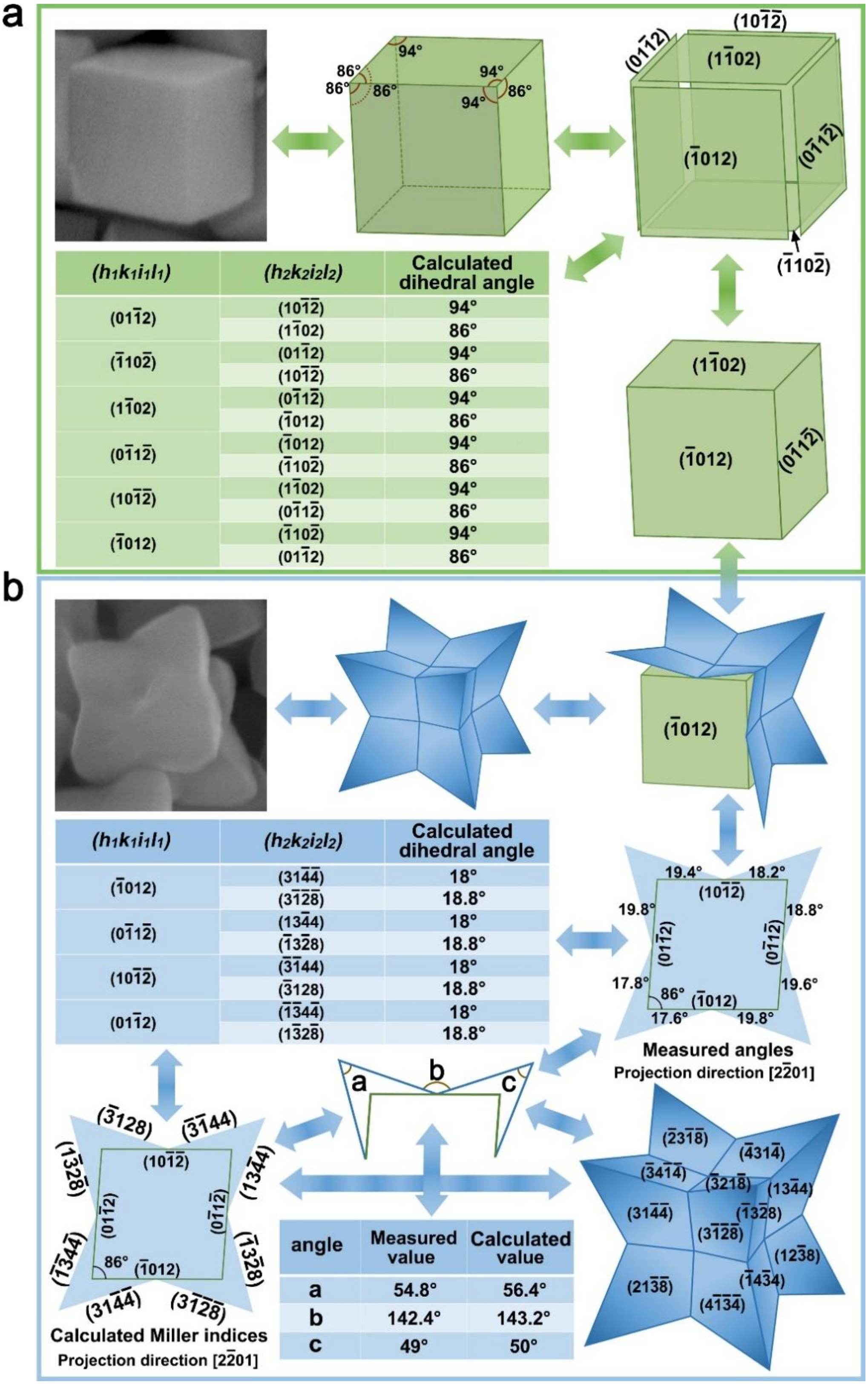

Fig. S4 Analytical geometrical models and their calculated dihedral angles of (**a**) P-Fe_2O_3 and (**b**) C-Fe_2O_3

As shown in Fig. S4a, P-Fe_2O_3 was enclosed by six $\{01\bar{1}2\}$ facets, coinciding with some previous reports [S9-S12]. The dihedral angles between two crystal facets were calculated by their Miller indices using the following formula:

$$\cos\theta = \frac{h_1h_2+k_1k_2+\frac{1}{2}(h_1k_2+h_2k_1)+\frac{3a^2}{4c^2}l_1l_2}{\sqrt{(h_1{}^2+k_1{}^2+h_1k_1+\frac{3a^2}{4c^2}l_1{}^2)(h_2{}^2+k_2{}^2+h_2k_2+\frac{3a^2}{4c^2}l_2{}^2)}} \qquad \text{(S1)}$$

Where θ is the included angle between two crystal planes, which is not equivalent to the dihedral angle. ($h_1k_1l_1$) and ($h_2k_2l_2$) are the Miller indices of two crystal planes.

According to the analysis of the two-dimensional top view observed along the direction $[2\bar{2}01]$ (Figs.1b and S4b), it could infer that C-Fe_2O_3 were evolved from P-Fe_2O_3 with smaller size [S11]. Within the range of approximate error permitting, the Miller indices of crystallographic planes could be deduced based on the projection angles. In comparison of the measured values and calculated values of angles projected along the direction $[2\bar{2}01]$, the eight side facets projected edge-on (Figs.1b and S4b) could be indexed as four $\{13\bar{4}4\}$ and four$\{12\bar{3}8\}$ crystallographic planes. The values of the projected angles were also calculated with the help of Eq. (S1). In addition, the measured values of angles a, b, and c coincided with the calculated values within the permissible range of error, which further proved the above analyses. Ulteriorly, according to the equivalent sides of the trigonal pyramid, three adjacent facets sharing one vertex out of twenty-four facets of C-Fe_2O_3 were identified as equivalent $\{13\bar{4}4\}$ or $\{12\bar{3}8\}$ crystallographic planes. In conclusion, C-Fe_2O_3 were enclosed by twelve equivalent $\{13\bar{4}4\}$ and twelve equivalent $\{12\bar{3}8\}$ facets as shown by an ideal geometrical model in Fig. S4b.

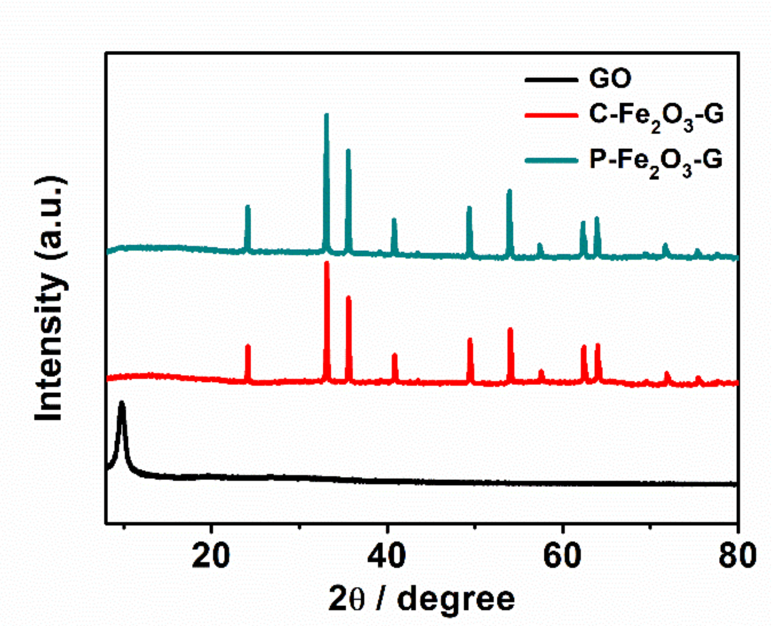

Fig. S5 XRD patterns of C-Fe_2O_3-G, P-Fe_2O_3-G and GO

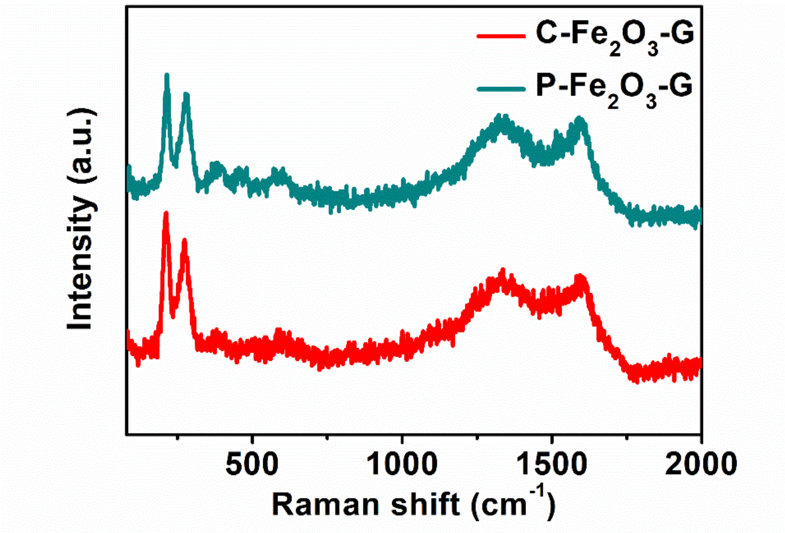

Fig. S6 Raman spectra of C-Fe_2O_3-G and P-Fe_2O_3-G

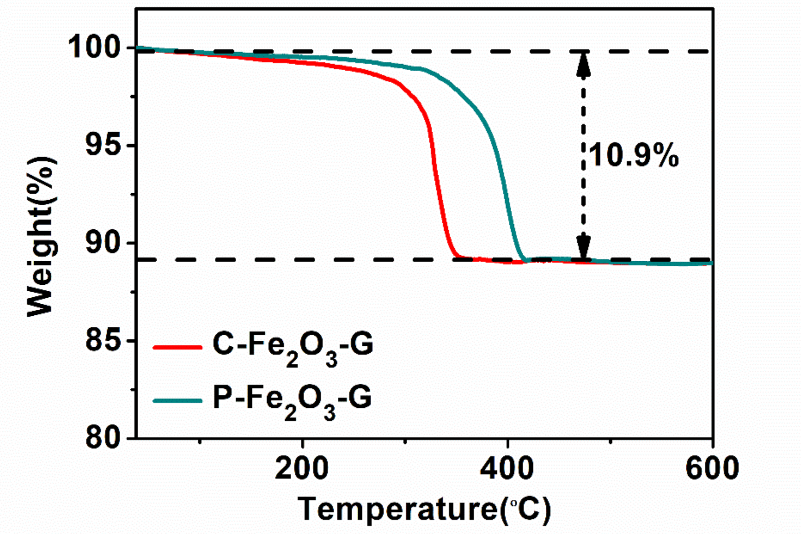

Fig. S7 TGA curve of C-Fe_2O_3-G and P-Fe_2O_3-G. The oxidation decomposition temperature of G in C-Fe_2O_3-G was lower than that of P-Fe_2O_3-G, which may be attributed to the higher catalytic oxidation activity of C-Fe_2O_3 [S10]

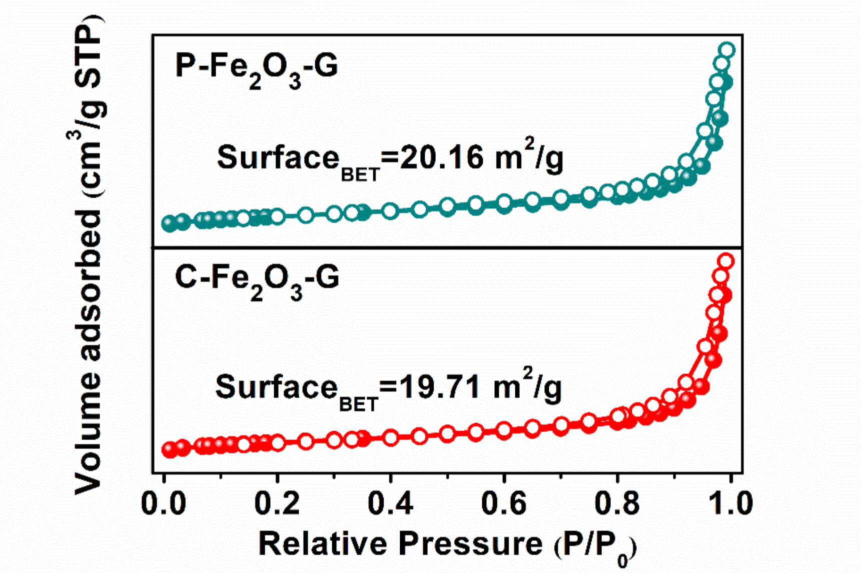

Fig. S8 N_2 adsorption-desorption isotherms of C-Fe_2O_3-G and P-Fe_2O_3-G

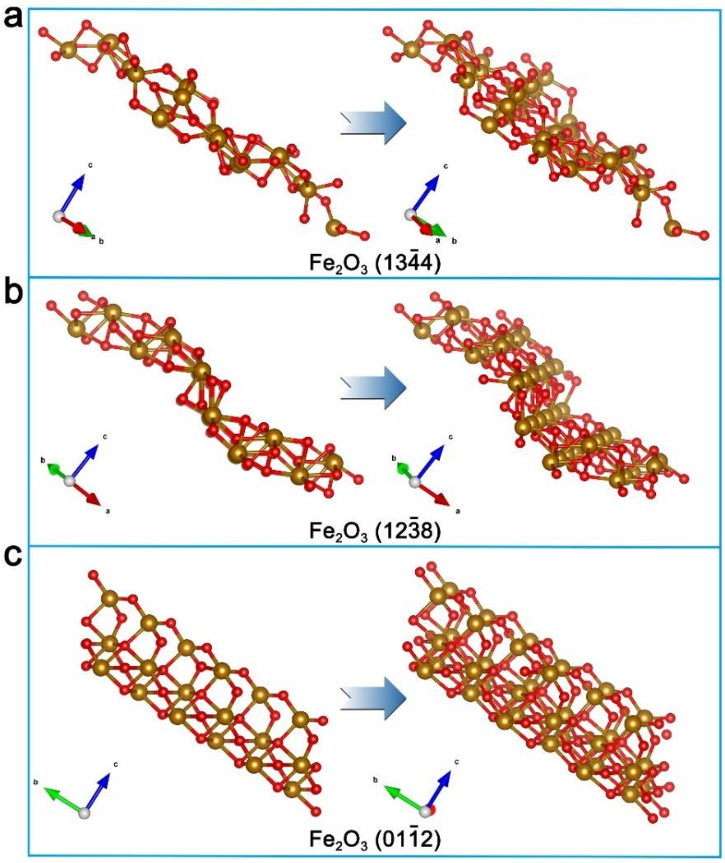

Fig. S9 Surface atom arrangement of Fe_2O_3 (**a**) $(13\bar{4}4)$, (**b**) $(12\bar{3}8)$ and (**c**) $(01\bar{1}2)$ facets

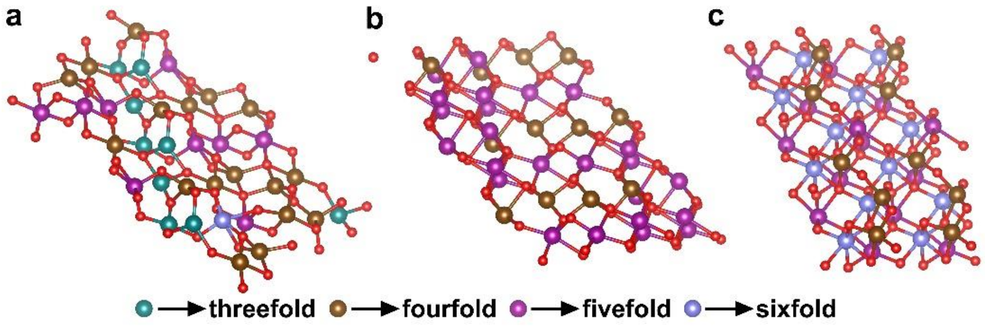

Fig. S10 Coordination model of Fe atoms on Fe_2O_3 (**a**) $(13\bar{4}4)$, (**b**) $(12\bar{3}8)$ and (**c**) $(01\bar{1}2)$ facets

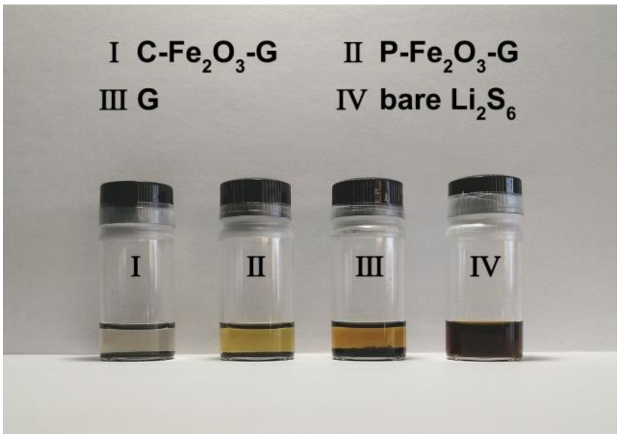

Fig. S11 Optical photograph of a bare Li_2S_6 solution and the Li_2S_6 solutions with different materials after static adsorption for 9 h

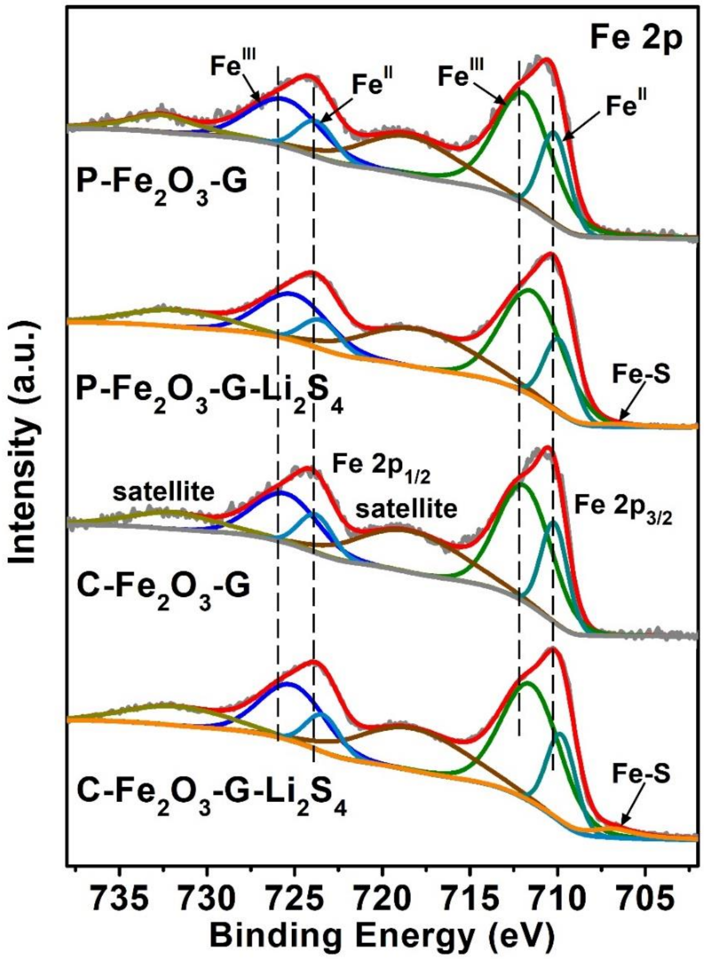

Fig. S12 Fe 2p XPS comparative analysis of C-Fe_2O_3-G and P-Fe_2O_3-G before and after interacting with Li_2S_4

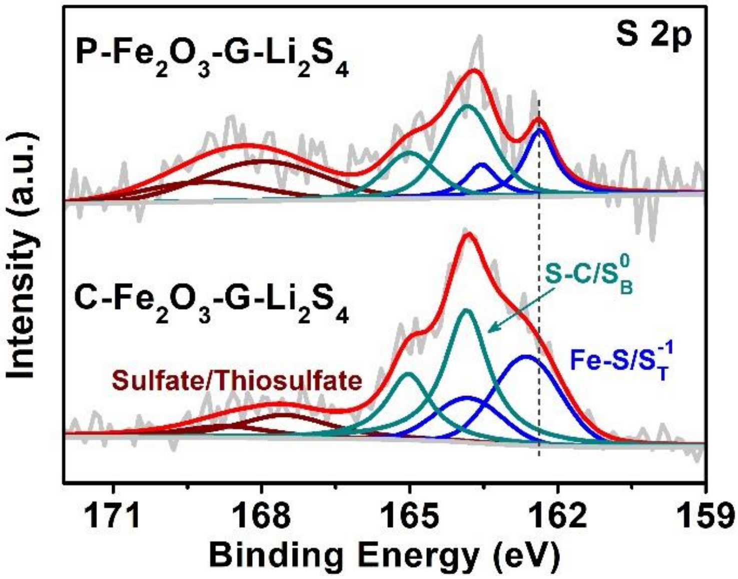

Fig. S13 S 2p XPS comparative analysis of C-Fe_2O_3-G and P-Fe_2O_3-G after interacting with Li_2S_4 (the bridging sulfur donated as S_B^0 and the terminal sulfur donated as S_T^{-1})

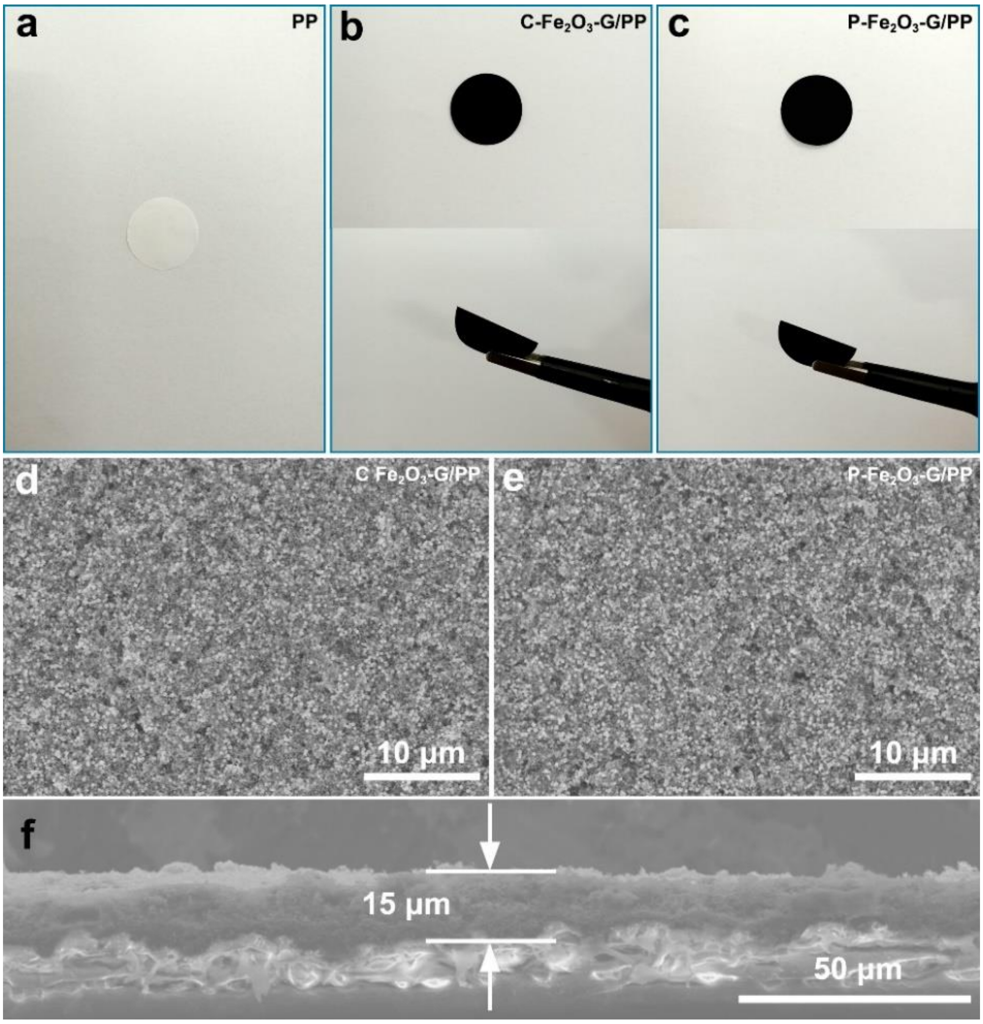

Fig. S14 Photos of (**a**) bare PP separator, (**b**) C-Fe_2O_3-G/PP separator and (**c**) P-Fe_2O_3-G/PP separator. SEM images of (**d**) C-Fe_2O_3-G interlayer and (**e**) P-Fe_2O_3-G interlayer. (**f**) Cross-section SEM image of the C-Fe_2O_3-G/PP separator

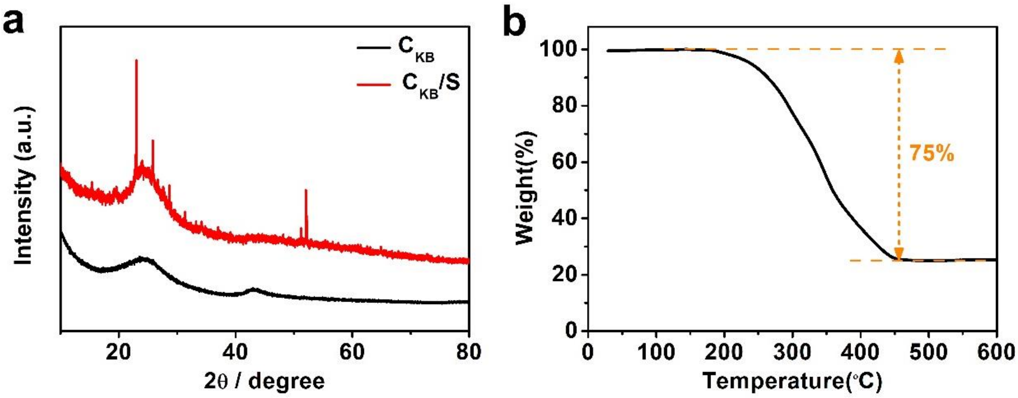

Fig. S15 (**a**) XRD patterns of Ketjen black carbon (C_{KB}) and C_{KB}/S composite. (**b**) TGA curve of C_{KB}/S composite

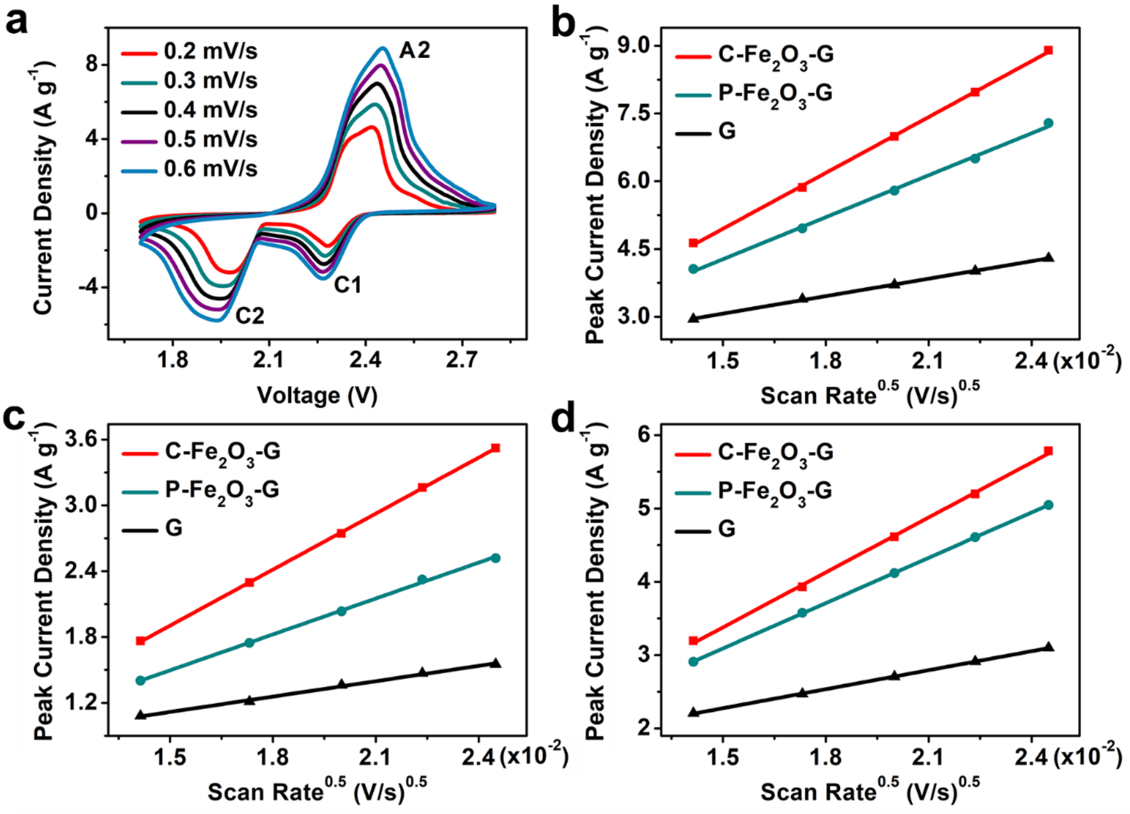

Fig. S16 (**a**) CV curves of the cell with C-Fe_2O_3-G interlayer recorded at various scan rates. Peak current density of (**b**) A2, (**c**) C1 and (**d**) C2 for cells with different interlayers vs. the square root of the scan rates

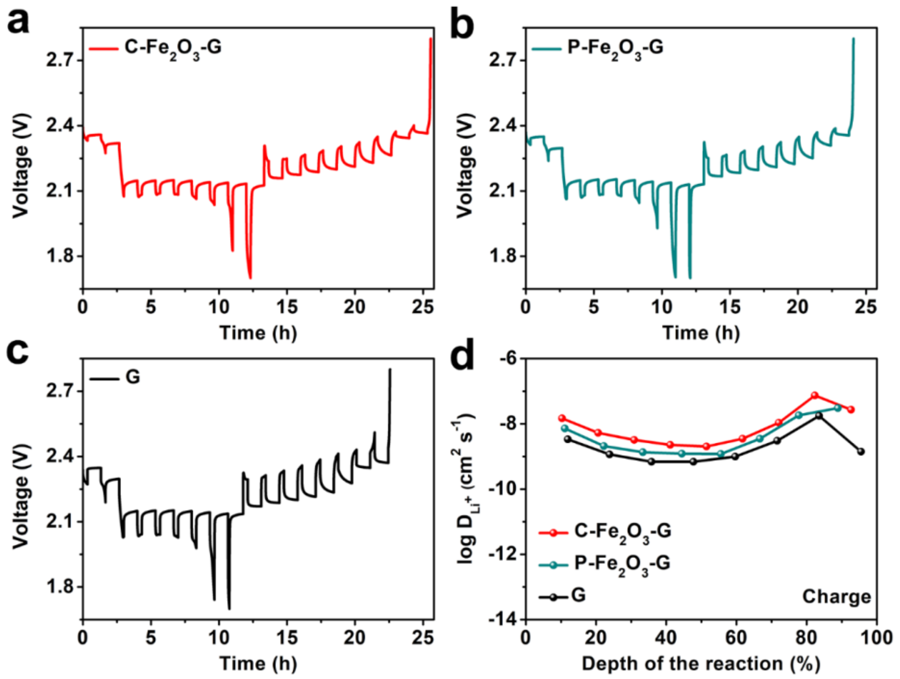

Fig. S17 GITT curves of (**a**) C-Fe_2O_3-G cell, (**b**) P-Fe_2O_3-G cell and (**c**) G cell. (**d**) The diffusion rate of Li^+ in different cells at charge process

The galvanostatic intermittent titration technique (GITT) was tested using the Neware battery test system in the voltage window of 1.7~ 2.8 V at 0.2 C. All the batteries were performed for 20 min interlarding with a 60 min relaxing time. According to the GITT profiles, lithium-ion diffusion coefficient (D_{Li}^{+}) could be calculated with the Eq. (S2):

$$D = \frac{4l^2}{\pi\tau}\left(\frac{\Delta E_S}{\Delta E_t}\right)^2 \qquad \text{(S2)}$$

where D is the ion diffusion coefficient, *l* represents the thick of the cathode, τ corresponds to current pulse (s). $\triangle Es$ refers to the steady-state potential change, $\triangle Et$ signifies the potential change during the constant current pulse including IR-drop (V).

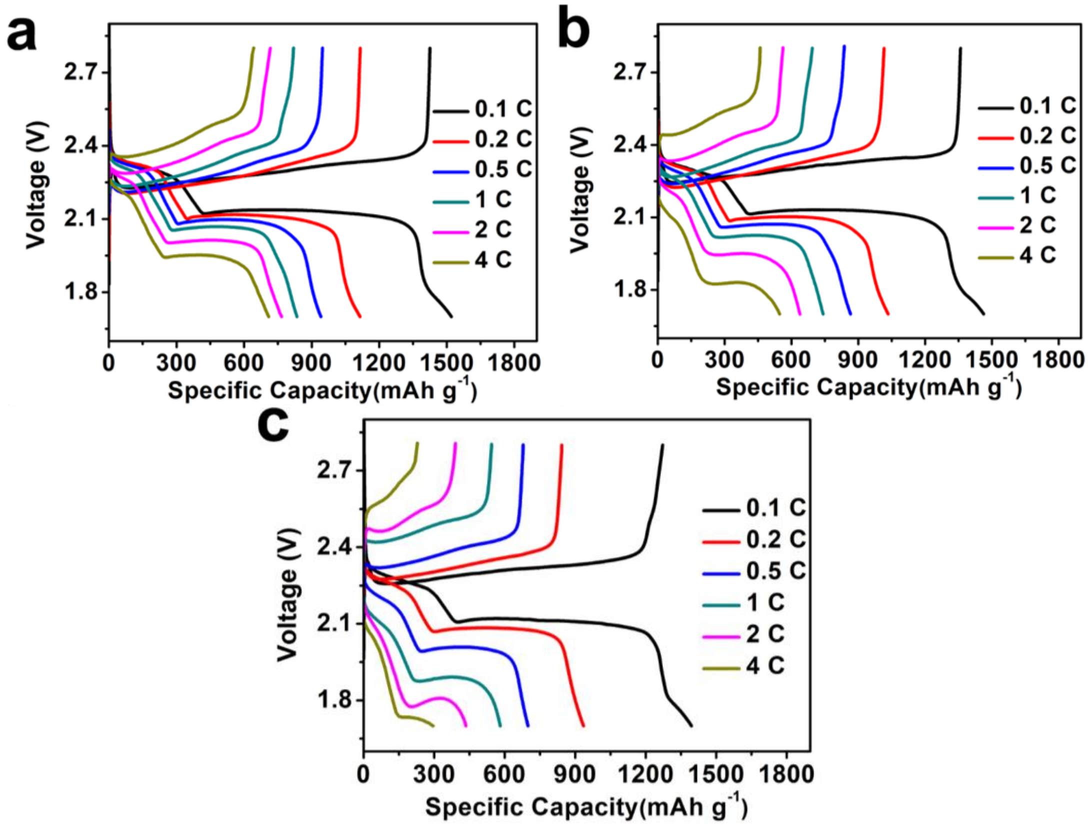

Fig. S18 The galvanostatic charge-discharge profiles of the (**a**) C-Fe_2O_3-G, (**b**) P-Fe_2O_3-G and (**c**) G cells at different current densities

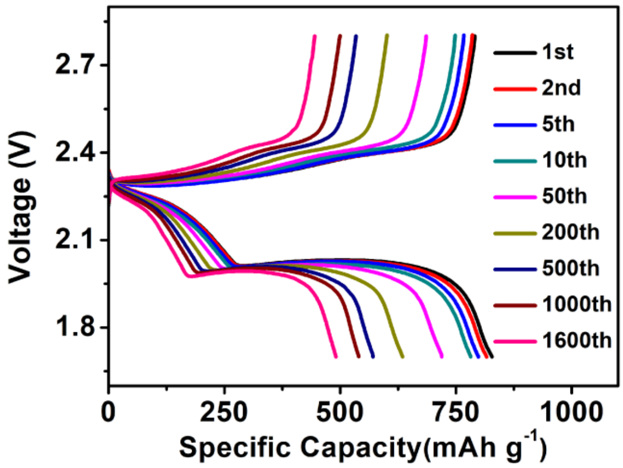

Fig. S19 The galvanostatic charge-discharge profiles of C-Fe_2O_3-G cell of different cycles at 2 C

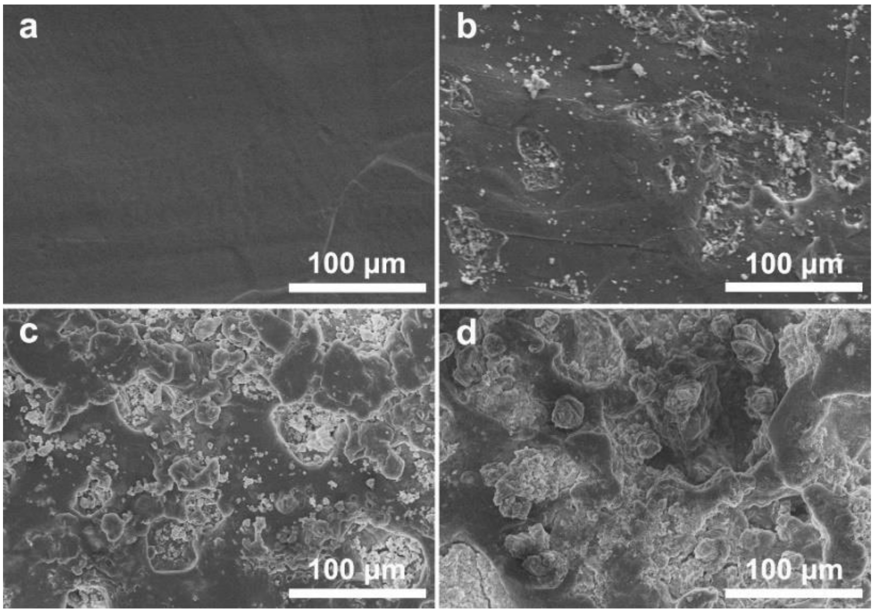

Fig. S20 SEM images of (**a**) original Li anode and Li anode after 500 cycles at 2 C with (**b**) C-Fe_2O_3-G, (**c**) P-Fe_2O_3-G and (**d**) G interlayers, respectively

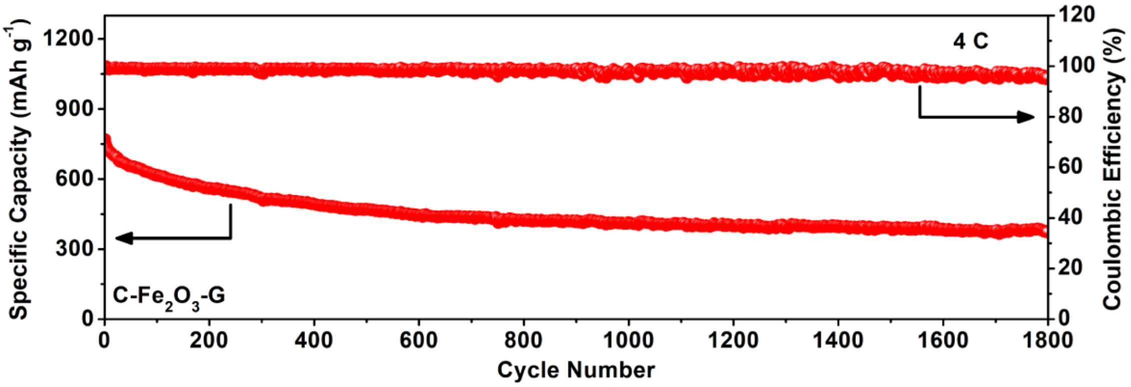

Fig. S21 Long-term cycle performance of C-Fe_2O_3-G cell at 4.0 C

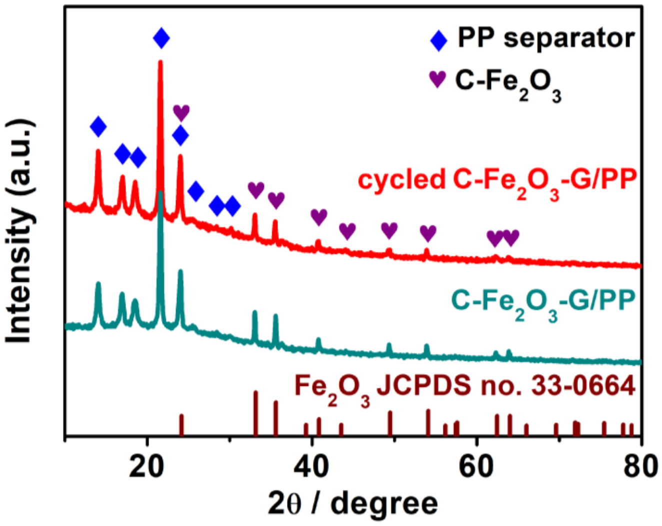

Fig. S22 XRD patterns of C-Fe_2O_3-G/PP and cycled C-Fe_2O_3-G/PP. The cycled C-Fe_2O_3-G/PP was washed thoroughly with toluene and DME to remove sulfur and other residues

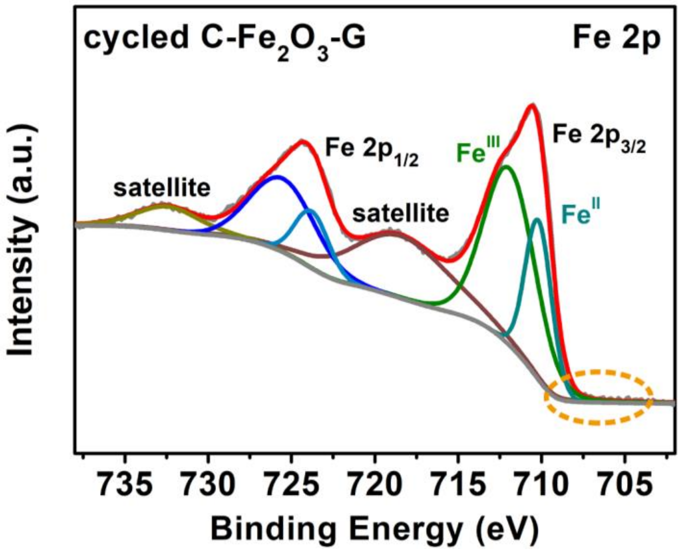

Fig. S23 Fe 2p XPS spectrums of the cycled C-Fe_2O_3-G. The cycled C-Fe_2O_3-G was washed thoroughly with toluene and DME to remove sulfur and other residues

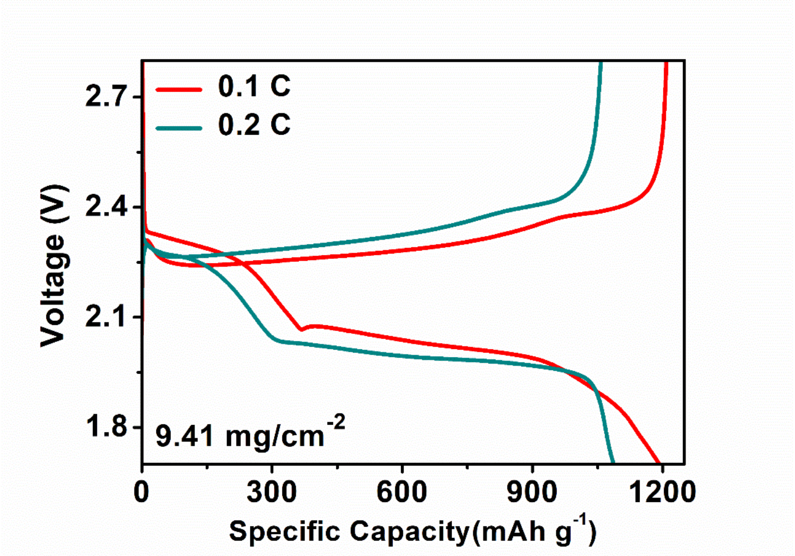

Fig. S24 Galvanostatic charge/discharge profiles of C-Fe_2O_3-G cell with high sulfur loading of 9.41 mg cm^{-2} at 0.1 and 0.2 C

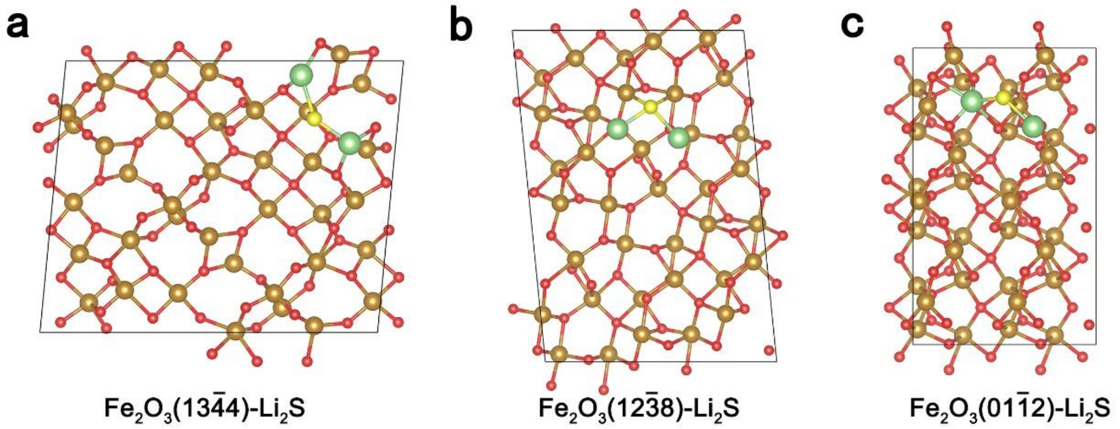

Fig. S25 Top sectional views of optimized geometries of Li_2S adsorbed on different Fe_2O_3 crystal faces

Table S1 Comparison of electrochemical performance of C-Fe_2O_3-G with various Fe-based oxide materials reported in previous works

materials	Initial Capacity (mAh g^{-1})	High Rate	Life Span	Capacity decay rate (per cycle)	Refs.
C-Fe_2O_3-G	1521	4 C	1600 cycle	0.025%	This work
YSC@Fe_3O_4	1366	2 C	200 cycle	0.074%	Adv. Mater. 2017 [S13]
Fe_3O_4/HPC	1424	2 C	1000 cycle	0.083%	Nano Energy 2021 [S14]
Fe_3O_4-NC	1316	4 C	1000 cycle	0.03%	Adv. Funct. Mater. 2019 [S15]
FeOOH	1449	2 C	500 cycle	0.05%	Small 2020 [S16]
Fe_3O_4/t-C_3N_4	1255	4 C	1000 cycle	0.024%	Nano-Micro Lett. 2020 [S17]
Fe_3O_4-PNCT	1375	5 C	1000 cycle	0.034%	Chem. Eng. J. 2021 [S18]
Fe_3O_4/CNSs	1225	5 C	1000 cycle	0.027%	J. Mater. Chem. A. 2020 [S19]
Fe_3O_4@void@C	1360	1 C	500 cycle	0.078%	Nanoscale, 2021 [S20]
CoNi1/3Fe_2O_4 @CNT	1332	2 C	250 cycle	0.063%	ACS Appl. Mater. Interfaces 2019 [S21]

Supplementary References

[S1] Y. Song, W. Zhao, N. Wei, L. Zhang, F. Ding et al., In-situ PECVD-enabled graphene-V_2O_3 hybrid host for lithium-sulfur batteries. Nano Energy **53**, 432-439 (2018). https://doi.org/10.1016/j.nanoen.2018.09.002

[S2] M. Wang, L. Fan, X. Sun, B. Guan, B. Jiang et al., Nitrogen doped $CoSe_2$ as a bifunctional catalyst for high areal capacity and lean electrolyte of Li-S battery. ACS Energy Lett. **5**(9), 3041-3050 (2020). https://doi.org/10.1021/acsenergylett.0c01564

[S3] P. Giannozzi, S. Baroni, N. Bonini, M. Calandra, R. Car et al., QUANTUM ESPRESSO: a modular and open-source software project for quantum simulations of materials. J. Phys. Condens. Matter **21**, 395502 (2009). https://doi.org/10.1088/0953-8984/21/39/395502

[S4] G. Kresse, D. Joubert, From ultrasoft pseudopotentials to the projector augmented-wave

method. Phys. Rev. B **59**, 1758-1775 (1999). https://doi.org/10.1103/PhysRevB.59.1758

[S5] P.E. Blöchl, Projector augmented-wave method. Phys. Rev. B **50**, 17953-17979 (1994). https://doi.org/10.1103/PhysRevB.50.17953

[S6] Y. Zhang, W. Yang, Comment on "generalized gradient approximation made simple". Phys. Rev. Lett. **80**, 890-890 (1998). https://doi.org/10.1103/PhysRevLett.80.890

[S7] J.P. Perdew, K. Burke, M. Ernzerhof, Generalized gradient approximation made simple. Phys. Rev. Lett. **77**, 3865-3868 (1996). https://doi.org/10.1103/PhysRevLett.77.3865

[S8] G. Henkelman, B.P. Uberuaga, H. Jónsson, A climbing image nudged elastic band method for finding saddle points and minimum energy paths. J. Chem. Phys. **113**, 9901 (2000). https://doi.org/10.1063/1.1329672

[S9] G.S. Park, D. Shindo, Y. Waseda, T. Sugimoto, Internal structure analysis of monodispersed pseudocubic hematite particles by electron microscopy. J. Colloid Interf. Sci. **177**(1), 198-207 (1996). https://doi.org/10.1006/jcis.1996.0021

[S10] H. Liang, X. Jiang, Z. Qi, W. Chen, Z. Wu et al., Hematite concave nanocubes and their superior catalytic activity for low temperature CO oxidation. Nanoscale **6**(13), 7199-7203 (2014). https://doi.org/10.1039/C4NR00552J

[S11] F. Gao, R. Liu, J. Yin, Q. Lu, Synthesis of polyhedral iron oxide nanocrystals bound by high-index facets. Sci. China Chem. **57,** 114-121 (2014). https://doi.org/10.1007/s11426-013-4973-y

[S12] J. Ouyang, J. Pei, Q. Kuang, Z. Xie, L. Zheng, Supersaturation-controlled shape evolution of α Fe_2O_3 nanocrystals and their facet-dependent catalytic and sensing properties. ACS Appl. Mater. Interfaces **6**(15), 12505-12514 (2014). https://doi.org/10.1021/am502358g

[S13] J. He, L. Luo, Y. Chen, A. Manthiram, Yolk-shelled C@Fe_3O_4nanoboxes as efficient sulfur hosts for high-performance lithium-sulfur batteries. Adv. Mater. **29**(34), 1702707 (2017). https://doi.org/10.1002/adma.201702707

[S14] Z. Zhao, Z. Yi, H. Li, R. Pathak, Z. Yang et al., Synergetic effect of spatially separated dual co-catalyst for accelerating multiple conversion reaction in advanced lithium sulfur batteries. Nano Energy **81**, 105621 (2021). https://doi.org/10.1016/j.nanoen.2020.105621

[S15] K. Lu, H. Zhang, S. Gao, H. Ma, J. Chen et al., Manipulating polysulfide conversion with strongly coupled Fe_3O_4 and nitrogen doped carbon for stable and high capacity lithium-sulfur batteries. Adv. Funct. Mater. **29**(4), 1807309 (2019). https://doi.org/10.1002/adfm.201807309

[S16] B. Wei, C. Shang, X. Wang, G. Zhou, Conductive FeOOH as multifunctional interlayer for superior lithium-sulfur batteries. Small **16**(34), 2002789 (2020). https://doi.org/10.1002/smll.202002789

[S17] S. Kim, S. Shirvani-Arani, S. Choi, M. Cho, Y. Lee, Strongly anchoring polysulfides by hierarchical Fe_3O_4/C_3N_4 nanostructures for advanced lithium-sulfur batteries. Nano-Micro Lett. **12**, 139 (2020). https://doi.org/10.1007/s40820-020-00475-5

[S18] S. Xin, J. Li, H. Cui, Y. Liu, H. We et al., Self-templating synthesis of prismatic-like N-doped carbon tubes embedded with Fe_3O_4 as a high-efficiency polysulfide-anchoring-conversion mediator for high performance lithium-sulfur batteries. Chem. Eng. J. **410**, 128153 (2021). https://doi.org/10.1016/j.cej.2020.128153

[S19] Z. Su, M. Chen, Y. Pan, Y. Liu, H. Xu et al., Expediting polysulfide catalytic conversion for lithium-sulfur batteries via in situ implanted ultrafine Fe_3O_4 nanocrystals in carbon nanospheres. J. Mater. Chem. A **8**(45), 24117-24127 (2020). https://doi.org/10.1039/D0TA08289A

[S20] T. Zhou, Z. Shen, Y. Wu, T. Han, M. Zhu et al., A yolk-shell Fe_3O_4 @void@carbon nanochain as shuttle effect suppressive and volume-change accommodating sulfur host for long-life lithium-sulfur batteries. Nanoscale **13**(16), 7744-7750 (2021). https://doi.org/10.1039/D1NR00658D

[S21] T. Liu, S. Sun, J. Hao, W. Song, Q. Niu et al., Reliable interlayer based on hybrid nanocomposites and carbon nanotubes for lithium-sulfur batteries. ACS Appl. Mater. Interfaces **11**(17), 15607-15615 (2019). https://doi.org/10.1021/acsami.9b02136
